# Magneto-optical Kerr effect in an A-type antiferromagnet

**DOI:** 10.1038/s41467-026-72577-4

**Published:** 2026-05-12

**Authors:** Veronika Sunko, Salman Ahsanullah, Vivek Jain, Sophie Weber, Sivaloganathan Kumaran, Jiaqiang Yan, Joseph Orenstein, Dmitry Ovchinnikov

**Affiliations:** 1 Department of Physics, University of California, Berkeley, CA USA; 2 Materials Science Division, Lawrence Berkeley National Laboratory, Berkeley, CA USA; 3 Institute of Science and Technology Austria, Klosterneuburg, Austria; 4 Department of Physics and Astronomy, University of Kansas, Lawrence, KS US; 5 Department of Physics and Astronomy, Chalmers University of Technology, Göteborg, Sweden; 6 Cavendish Laboratory, University of Cambridge, Cambridge, UK; 7 Materials Science and Technology Division, Oak Ridge National Laboratory, Oak Ridge, TN USA

**Keywords:** Magnetic properties and materials, Condensed-matter physics

## Abstract

Magneto-optic Kerr effect (MOKE) is a powerful probe of broken time-reversal symmetry (${{{\mathcal{T}}}}$), typically used to study ferromagnets. While MOKE has been observed in some antiferromagnets (AFMs) with vanishing magnetization, it is often associated with structures whose symmetry is lower than basic collinear, bipartite order. In contrast, theory predicts a mechanism for MOKE intrinsic to all AFMs of A-type, i.e. layered AFMs in which ferromagnetic layers are antiferromagnetically aligned. Here we report the experimental confirmation of this mechanism in a bulk AFM. We achieve this by measuring the imaginary component of MOKE as a function of photon energy in MnBi_2_Te_4_, an A-type AFM where ${{{\mathcal{T}}}}$ is preserved in combination with a translation, and comparing the experimental results with model calculations. Our model suggests that observable MOKE should be expected in all collinear A-type AFMs with out-of-plane spin order, thus enabling optical detection of AFM domains and expanding the scope of MOKE to few-layer AFMs.

## Abstract (web summary)

The magneto-optical Kerr effect, MOKE, is a bread-and-butter technique for studying ferromagnets. While some antiferromagnetic materials have a finite MOKE signal, this is typically related to reduced symmetry or noncollinear magnetic order. Here, Sunko and coauthors find a finite MOKE signal in a bulk A-type layered antiferromagnet, MnBi2Te4.

## Introduction

The detection of broken time-reversal symmetry (${{{\mathcal{T}}}}$) in quantum materials is of fundamental interest and practical relevance. Recent advances in the synthesis and exfoliation of van der Waals magnets have highlighted the critical role of optical techniques in detecting ${{{\mathcal{T}}}}$-breaking in samples just a few atomic layers thick^1–9^, whose small mass poses a challenge for bulk probes of magnetism. The magneto-optic Kerr effect (MOKE), which is the difference in reflectivity for left and right circularly polarized (LCP and RCP) light, is routinely used for detection of ferromagnetic (FM) order. Which classes of antiferromagnetic (AFM) order yield a measurable MOKE signal, and the underlying mechanism in each case, are questions of long-standing fascination. Here we show that MOKE is a more powerful and general probe of AFM order than previously thought, with implications for research and applications of both bulk and few-layer AFMs.

**Fig. 1:**
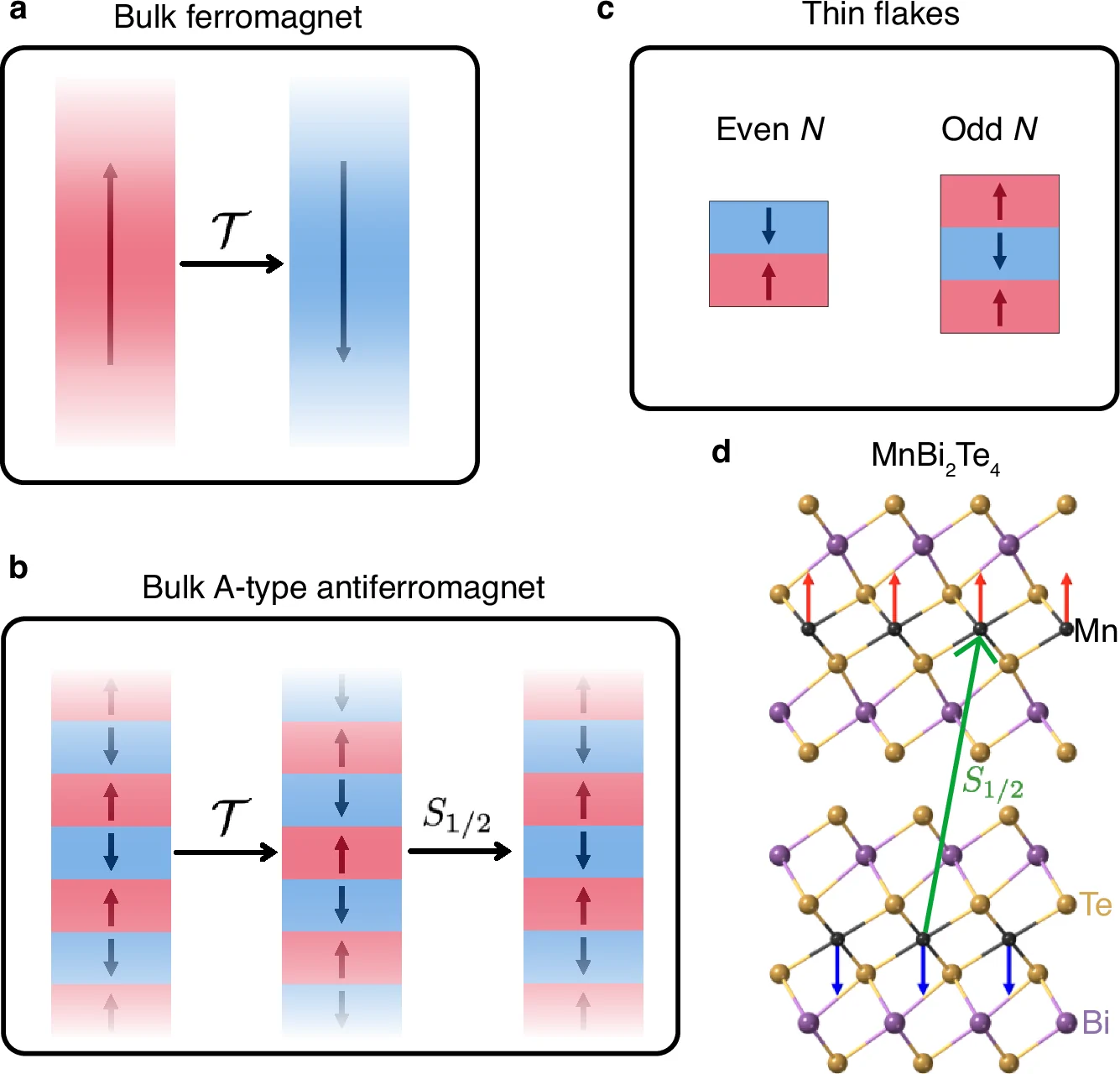
Schematics of representative magnetic structures. **a** Ferromagnet, where ${{{\mathcal{T}}}}$ reverses the sign of the order parameter; **b** A-type antiferromagnet, where the sign reversal by ${{{\mathcal{T}}}}$ can be undone by a translation by half a magnetic unit cell (*S*_1/2_); **c** Thin flakes of an A-type antiferromagnet: flakes of odd layer number *N* have a net magnetic moment, while those with even *N* do not. **d** Two layers of the MnBi_2_Te_4_ crystal and magnetic structure, demonstrating the A-type AFM phase. Opposite spins are related by *S*_1/2_.

Time reversal flips the direction of spin, turning a ferromagnet into its time-reversed counterpart (Fig. 1a). The two configurations with opposite magnetization *M*, called domains, enable the storage, processing, and retrieval of information in the form of a classical 0 or 1. Antiferromagnetic (AFM) order is also characterized by degenerate ground states related by ${{{\mathcal{T}}}}$, but *M* = 0 in each domain. In the simplest example neighboring spins are antiparallel (Fig. 1b). AFMs play an integral role in critical technologies^10,11^, and interest in their properties has continued to grow^12–15^. Zero magnetization can be a feature, as the absence of the long-range magnetic dipolar interaction leads to enhanced scalability.

A non-zero *M* induces a difference in the index of refraction of LCP and RCP light, yielding a mechanism for MOKE that is essentially the same in all FMs. MOKE is therefore a reliable technique for distinguishing FM domains with high-spatial resolution. In contrast, domains have been detected through MOKE only in a few AFMs^16–18^, and the mechanism that underlies this signal has been attributed to specific properties of individual compounds.

Here, we focus on a class of AFMs in which the time-reversed states are related by translation by half the magnetic unit cell, *S*_1/2_, illustrated in bulk and thin flake forms in Fig. 1b, c, respectively. We choose this class because the product ${{{\mathcal{T}}}}{S}_{1/2}$ ensures that the index of refraction of LCP and RCP light is the same as they propagate through an unbounded medium, a condition that at first sight seems to stipulate the absence of MOKE, since it is the difference of the two indices that causes MOKE in FMs. However, this conclusion is incorrect: MOKE by its nature involves a bounding surface that breaks ${{{\mathcal{T}}}}{S}_{1/2}$, unless *S*_1/2_ is parallel to the surface. Therefore, ${{{\mathcal{T}}}}{S}_{1/2}$ imposes no constraints on MOKE at surfaces that break translational invariance described by *S*_1/2_.

We investigate MnBi_2_Te_4_, a layered topological AFM with a Néel temperature of 25 K^19^, as an example of a ${{{\mathcal{T}}}}{S}_{1/2}$ - symmetric AFM. Spins in individual Mn layers in MnBi_2_Te_4_ are parallel to each other, and antiparallel to the spins in neighboring layers (Fig. 1d). This order, called A-type, persists to the surface of MnBi_2_Te_4_^20^. MOKE was recently reported in the wavelength range of 500–1000 nm in thin flakes of MnBi_2_Te_4_, in samples with an odd number of layers (*N*), which have a net magnetization, and in samples with an even *N*, which do not^21–24^. While symmetry permits MOKE for all *N*, it does not identify the underlying mechanism.

Axion electrodynamics associated with topology was proposed as the mechanism for MOKE in MnBi_2_Te_4_^23,25^. The discontinuity in the topological ${{{{\mathcal{Z}}}}}_{2}$ invariant at the sample/vacuum interface manifests as a quantized surface Hall conductance, which is indistinguishable from a quantized trace of the magnetoelectric (ME) tensor in the static limit^26^. The surface conductance gives rise to a traceful, non-quantized ME tensor above zero frequency^25,27^. Optical phenomena associated with a traceful ME tensor (collectively known as axion electrodynamics^28^), provide a mechanism for MOKE^25,29,30^, as demonstrated in studies of the non-topological ME Cr_2_O_3_^16^.

A distinct mechanism for MOKE in ${{{\mathcal{T}}}}{S}_{1/2}$ symmetric AFMs was proposed by Dzyaloshinskii and Papamichail^31^. They treated the layered AFM as a stack of FMs with alternating spin direction. The dielectric tensor of each layer is assumed to be the same as in an unbounded medium with the same FM order, and MOKE arises due to the variation in dielectric properties as the wave propagates. In the alternating FM framework, the optical response arises from the bulk magnetic structure, and the surface conductance does not play a special role, unlike in the axion electrodynamics mechanism, where the surface contribution is essential. The alternating FM mechanism is allowed in all collinear AFMs whose spins are oriented perpendicular to the layers, regardless of the number of layers, band topology or symmetry. To the best of our knowledge, MOKE arising from the alternating FM effect has not been experimentally recognized prior to this work.

In this work we report measurements of MOKE in an optically thick sample of MnBi_2_Te_4_ ( ~ 136 nm thick, Fig. S3), which we refer to as infinite layer MnBi_2_Te_4_, as a function of magnetic field, *H*, wavelength of the optical probe, *λ*, and the position on the sample. We find that non-zero MOKE appears in the *M* = 0 state, and that its spectrum is quantitatively captured by the alternating FM model^31^, opening the door for MOKE studies of a broader class of AFMs than was previously considered feasible.

## Results

### The experiment: magnetic field, position, and wavelength dependence

The complex MOKE angle is defined by the relation, *Θ* ≡ − *iδr*/*r*, where the reflectivities of incident LCP and RCP light are given by *r* = *r*_0_ ± *δr* (the sign and polarization conventions are defined in the Methods section). Throughout this paper, we show measurements of the imaginary part of *Θ*, which is known as reflection circular dichroism (RCD). We measure RCD using a photoelastic modulator to vary the helicity of the incident light at 50 kHz, using a setup illustrated in Fig. S4. The change in reflectivity synchronous with the helicity modulation is proportional to RCD (see Methods for details of the experiment).

**Fig. 2:**
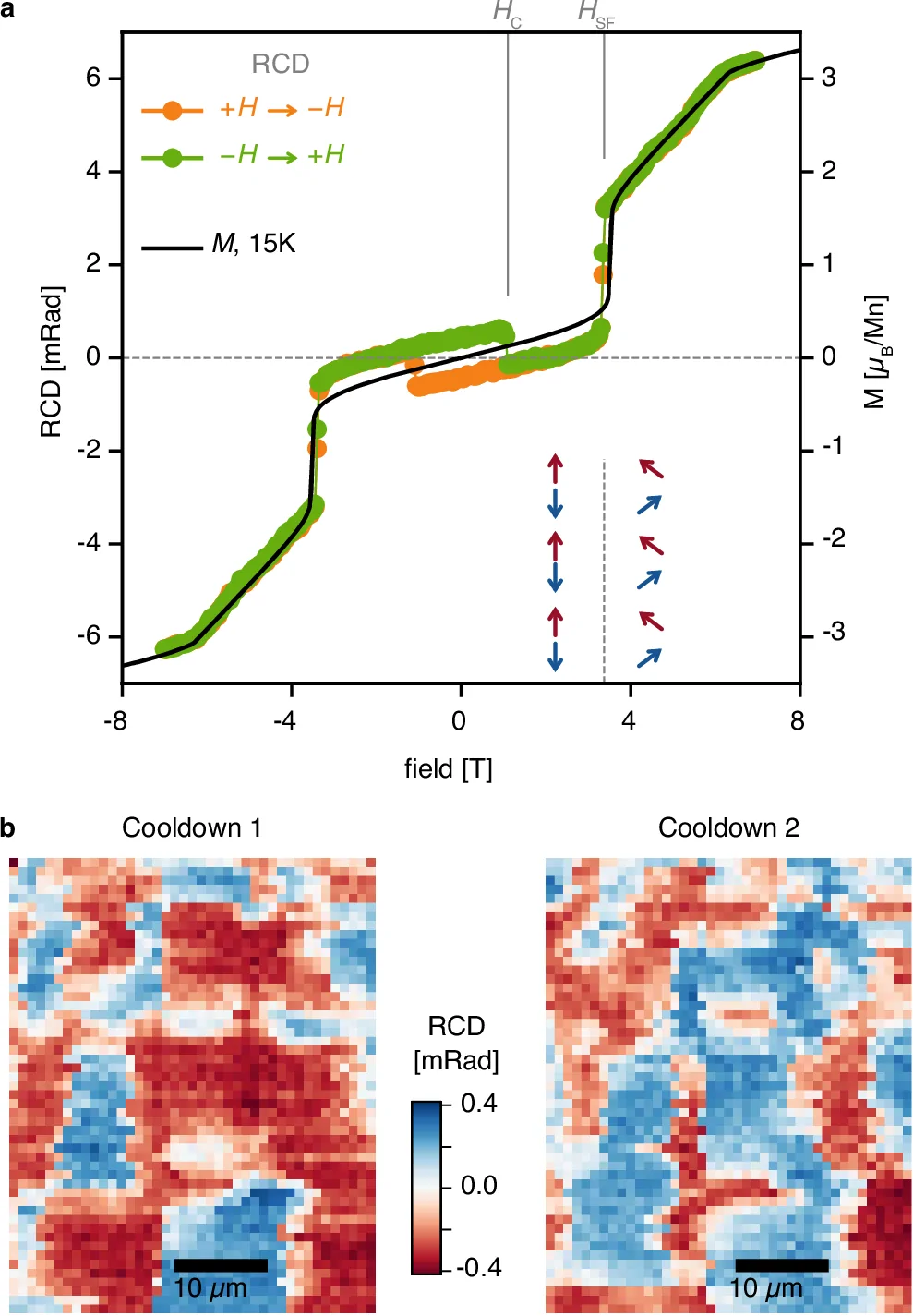
Magneto-optical measurements of MnBi_2_Te_4_. **a** Reflection circular dichroism (RCD) measured at 525 nm as a function of magnetic field, showing a jump at the spin-flop transition (*H*_*SF*_ ≈ 3.4 T) and a hysteresis loop with a coercive field of *H*_*C*_ ≈ 1 T. A small setup-induced background contribution has been subtracted (Fig. S7). RCD is compared with bulk magnetization measurements taken at 15 K with the field applied along the crystallographic *c*-axis (black line). The inset illustrates the bulk spin-flop transition. **b** Spatially resolved RCD measurements at *H* = 0, revealing AFM domain structures that change between cooldowns.

In Fig. 2a, we compare the magnetic field dependence of RCD to that of bulk magnetization at 15 K, measured on a sample from the same batch. RCD reveals two discontinuous features: a jump at *H*_*SF*_ ≈ 3.4 T and a hysteresis loop with a coercive field of *H*_*C*_ ≈ 1 T. The jump corresponds to a spin-flop (SF) transition, illustrated in the inset, which is also seen in magnetization. The corresponding magnetization increase is $\Delta {M}_{SF} \approx 1.2 {\mu }_{B}/{{{\rm{Mn}}}} \approx 0.4{M}_{s}$, where ${M}_{s}=3{\mu }_{B}/{{{\rm{Mn}}}}$ is the ordered moment at 15 K^32^. The hysteresis loop has no counterpart in bulk magnetization measurements, which find *M* = 0 for *H* < *H*_*SF*_. Therefore, the two non-zero values of RCD at *H* = 0 correspond to time-reversed states, each with *M* = 0. Further evidence for this interpretation is provided by spatially resolved measurements (Fig. 2b) that reveal spontaneous formation of domains with positive and negative RCD values in zero field, with distinct structures in different *H* = 0 cooldowns.

**Fig. 3:**
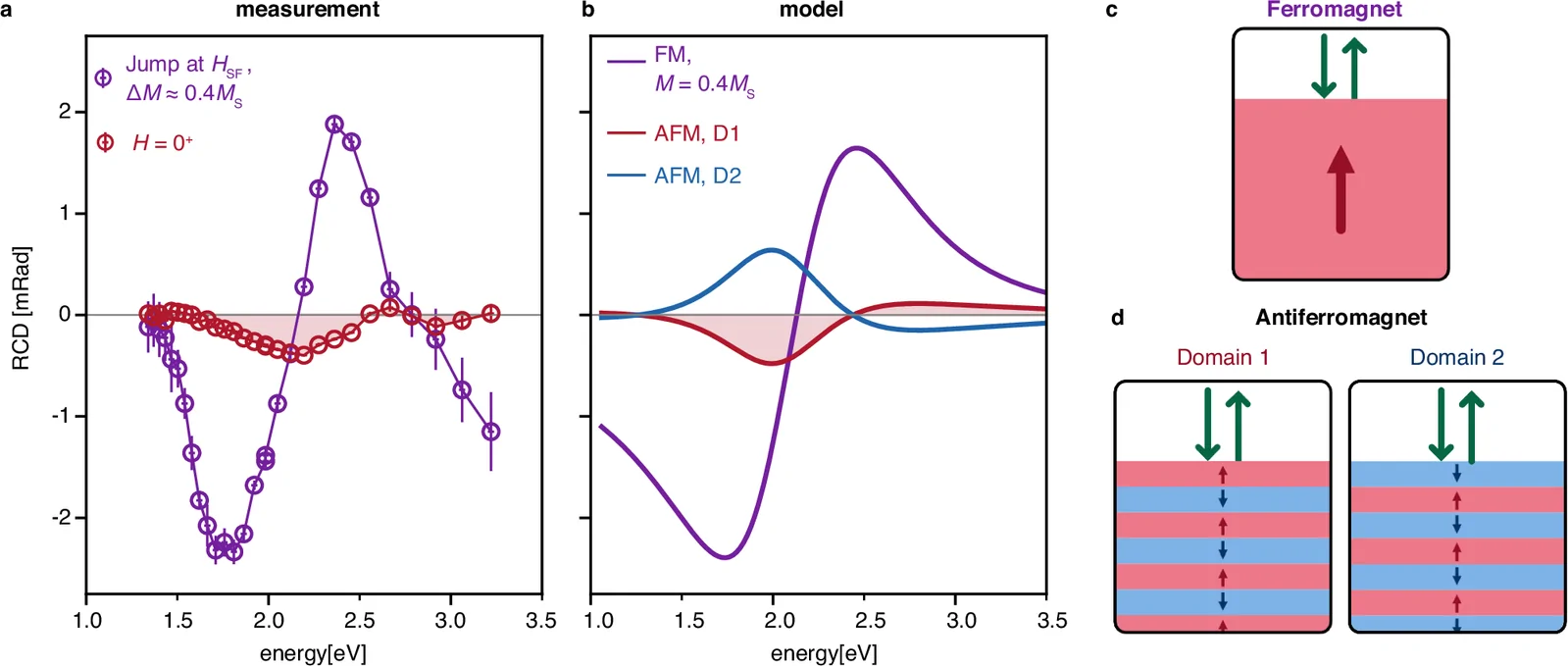
Spectroscopic signature of MOKE in antiferromagnetic MnBi_2_Te_4_. **a** Measured RCD spectrum at the spin-flop transition (purple) and at zero field (red), showing distinct spectral shapes. For each photon energy, we measured the magnetic field dependence of RCD (Fig. S8). The AFM spectrum is half of the difference between RCD on the downward (*H* = 0^+^) and upward (*H* = 0^−^) field sweep, averaged across the hysteresis loop, between  ± 0.9 T. The SF spectrum is given by Θ_*SF*_ = Θ(3.4T) − [Θ(3.2T) − Θ(0^+^)]. The error bars for the *H* = 0^+^ measurement represent the standard deviation across this field range. The larger error bars for the SF spectrum reflect the fact fewer measurements were used to extract the corresponding RCD value (*H* = 3.4 T and *H* = 3.2 T). Laser power fluctuations do not contribute to these error bars, since the RCD is normalized by the simultaneously measured reflectivity (the same detector voltage is demodulated at two frequencies). The only relevant systematic uncertainty is from the PEM calibration, which for our Hinds PEM-100 is ≤5%, corresponding to  < 0.1 mrad on the RCD scale. **b** RCD spectra computed from the Lorentz model (Eq. (1), with parameters from Table M1) for a ferromagnet (purple, schematic in **c**) and two antiferromagnetic domains (red and blue, schematics in **d**). The ferromagnetic RCD is scaled by a factor of 0.4 from that calculated for a saturated moment, to be directly comparable to the measured RCD jump at the spin-flop transition.

The existence of two discontinuities in RCD vs *H* provides an unusual opportunity to compare the MOKE spectrum in a phase with *M* ≠ 0, that is the spin-flop (SF) phase, and a ${{{\mathcal{T}}}}{S}_{1/2}$ -symmetric AFM (*M* = 0) phase in the same material. We measured the magnetic field dependence of RCD in the photon energy range from 1.34 eV (925 nm) to 3.22 eV (385 nm) (Fig. S8), and extracted RCD spectra in the two phases. In Fig. 3a, we compare the RCD spectrum corresponding to the SF transition with the RCD spectrum in the AFM phase. The AFM spectrum is half of the difference between RCD at *H* = 0^+^ and *H* = 0^−^, where *H* = 0^+^ and *H* = 0^−^ denote zero field as approached from positive and negative fields. The SF spectrum, corresponding to the jump in magnetization across the transition, is given by ${\Theta }_{SF}=\Theta (3.4\,{{{\rm{T}}}})-\left[\Theta (3.2\,{{{\rm{T}}}})-\Theta ({0}^{+})\right]$, because *Θ*(3.2 T) is a sum of the antiferromagnetic and magnetization contributions, while in the spin flop phase, at 3.4 T, there is no antiferromagnetic contribution.

The two spectra are strikingly different, ruling out a small uncompensated bulk ferromagnetic moment as a trivial explanation for RCD at *H* = 0. Further, in the SI (Fig. S2) we consider whether modified surface properties could account for this observation, and we conclude that such explanation is inconsistent with the data. This leaves us with the following conclusion: MOKE at *H* = 0 arises from the bulk antiferromagnetic structure. The fact that the two spectra are rich in features creates an opportunity to constrain theories: a suitable theoretical model should simultaneously reproduce both of them.

### Lorentz model: infinite layer and finite samples

The RCD spectrum measured in the SF state is consistent with resonant MOKE in a FM. Both the magnitude and zero crossing can be reproduced by a Lorentz oscillator whose resonant photon energy differs for LC and RC polarized light. The corresponding dielectric function is:

1 $${\varepsilon }_{\pm }(\omega )={\varepsilon }_{\infty }+\frac{f}{{({\omega }_{0}\pm \delta \omega )}^{2}-{\omega }^{2}-i\gamma \omega },$$

where *f*, *ω*_0_, and *γ* are oscillator strength, resonant frequency, and damping. These parameters, together with a background contribution, *ε*_*∞*_, are chosen to reproduce the measured optical conductivity in the spectral range of interest, reported in ref. ^33^. The good agreement between the measured quantities and those calculated from the Lorentz model is shown in Fig. S5. Finally, *δω*, the difference in resonance frequency for LC and RC polarized light, is chosen to fit the magnitude of the measured RCD. The purple curve in Fig. 3b shows that the single oscillator model captures the main features of the observed ferromagnetic RCD spectrum (Fig. 3a).

Our quantitative description of RCD in the ferromagnetic phase of MnBi_2_Te_4_ leads us to consider the alternating FM model for the RCD in its AFM state: a stack of FM layers in which the sign of *δω* alternates in neighboring layers. We use the transfer matrix formalism^34^, following the approach in ref. ^35^, to calculate the reflection coefficient from the stack. The number of layers is 100, and the thickness of each layer, *d* = 1.3 nm, corresponds to the separation between spins in MnBi_2_Te_4_. The simulated RCD spectra for the two ${{{\mathcal{T}}}}$-reversed AFM domains (Fig. 3d), are shown in red and blue in Fig. 3b. Remarkably, this minimal model captures the spectral shape and the magnitude of RCD in the AFM phase of MnBi_2_Te_4_, without introducing free parameters beyond those that describe the FM phase. In the SI we extend this analysis by using a dielectric function computed from a density functional theory (DFT) calculation to construct the alternating FM model, and we find that this approach also yields the correct magnitude of RCD in the AFM phase (Fig. S6). To the best of our knowledge, this is the first experimental evidence for an RCD arising from an alternating FM model in a bulk AFM.

**Fig. 4:**
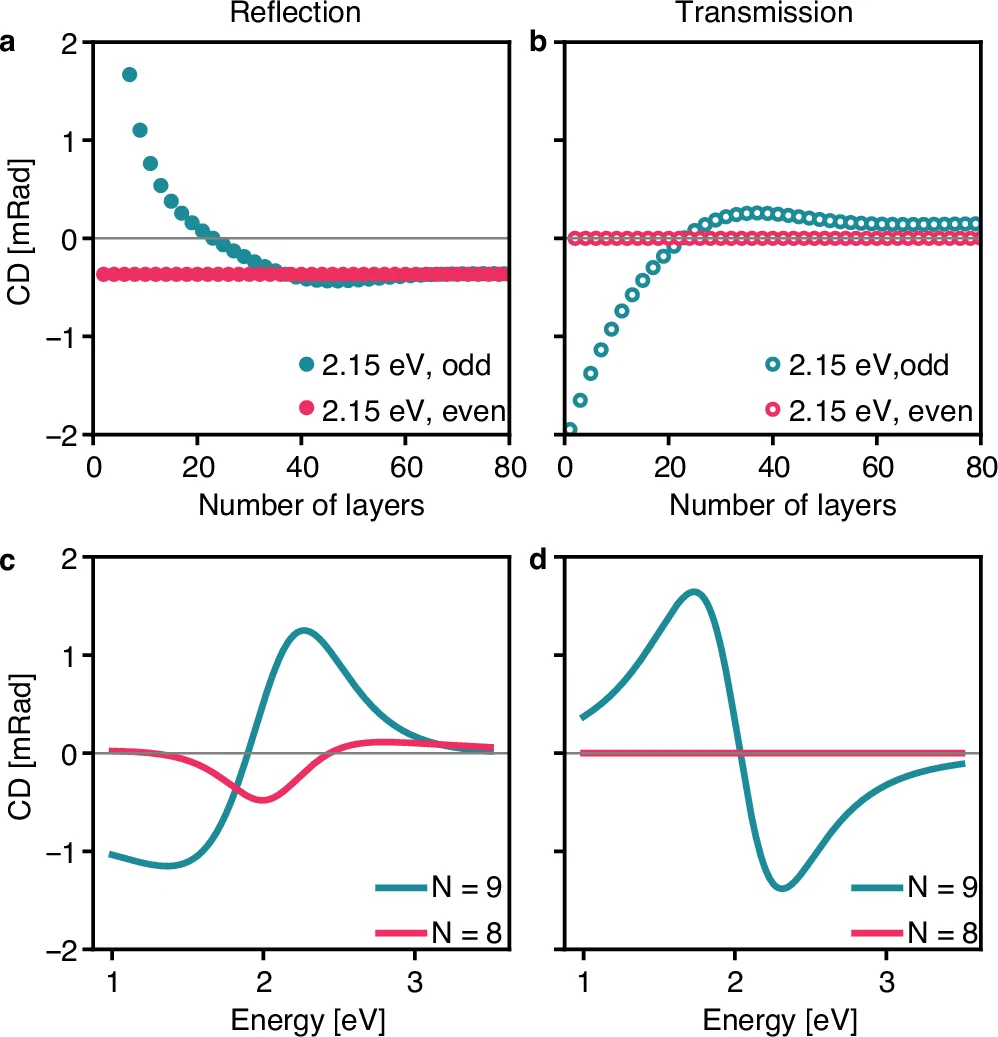
Dependence of circular dichroism on the number of layers. **a**,**b** Calculated reflection (RCD) and transmission (TCD) circular dichroism as a function of layer number for even and odd layer stacks, using the same model and parameters as in Fig. 3. **c**,**d** RCD and TCD spectra for 8-layer (8L) and 9-layer (9L) AFM stacks.

Next, we show that the key features of RCD and transmission circular dichroism (TCD) that are seen in few layer crystals in ref. ^23^ can be understood within the same model. We performed a series of revealing transfer matrix calculations, varying the number of layers while keeping the thickness of individual layers fixed. In Fig. 4a, b we plot the calculated RCD and TCD, respectively, as a function of layer number at fixed photon energy. We find that both RCD and TCD depend strongly on *N* if it is odd, but are independent of *N* when it is even, consistent with the findings of ref. ^31^. When the slab thickness *Nd* far exceeds the optical penetration depth, RCD in even and odd layer samples converge to the same value, as expected. TCD vanishes for all even layer stacks, a phenomenon that can be understood from a symmetry perspective: even layer stacks are symmetric under a product of inversion and time-reversal symmetry, which prohibits circular dichroism in transmission^23,36–38^.

Further, the dichroism spectra for even and odd layer stacks are strikingly different (Fig. 4c, d). For *N* = 8, the RCD spectrum is identical to the spectrum of the bulk AFM crystal (Fig. 3b), while spectrum for *N* = 9 resembles that of the bulk SF phase, as *M* ≠ 0 for odd layer stacks. The maximum RCD is about three times larger for *N* = 9 than for *N* = 8. However, these calculations assume that samples are suspended in vacuum, which is not a realistic experimental scenario. The transfer matrix approach allows us to include the substrate in the calculation of reflectivity^35^, and we find that doing so preferentially suppresses dichroism in odd-layer samples. In Fig. S9 we show that the maximum magnitude of RCD for samples on sapphire and diamond substrates, used in ref. ^23^, is approximately equal in magnitude for even and odd layer stacks, while they maintain the characteristic spectral shapes shown in Fig. 4c. We therefore find that the alternating FM model with realistic parameters captures the main experimental results on *N*-even and *N*-odd flakes of MnBi_2_Te_4_ reported in ref. ^23^, as well as on infinite layer flakes reported here.

### Bilayer: the exact solution and the physical picture

While the numerical results shown in the above section capture the experimental results, the underlying physics is somewhat mysterious, and poses the following questions: why does MOKE arise in ${{{\mathcal{T}}}}{S}_{1/2}$-symmetric AFMs within the alternating FM model, and what influences its magnitude? Why is its value independent of the (even) number of layers? Why does TCD vanish? To address these questions, we first consider the analytical solution for MOKE in a bilayer, and then offer an intuitive physical picture, shedding light on the experimental, numerical and analytical findings.

**Fig. 5:**
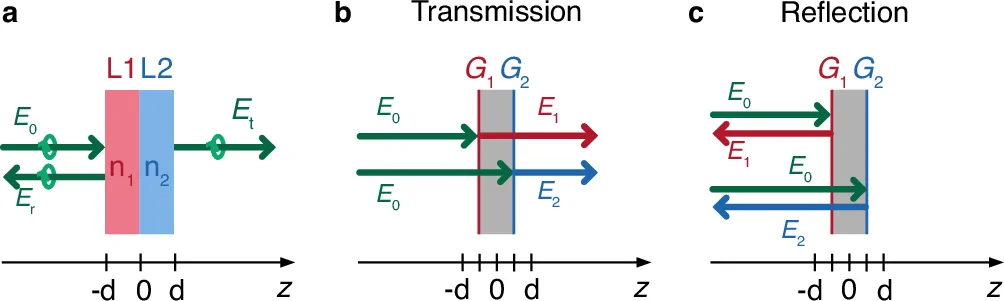
Illustration of the model for MOKE in an antiferromagnetic bilayer. **a** Schematic representation of a bilayer, consisting of two layers of indices of refraction *n*_1,2_ = *n* ± *δn* when probed with circularly polarized light. *δn* is induced by the opposite magnetization in the two layers,  ± *M*_*z*_. **b**,**c** Simplified representation of a bilayer, where the layers are replaced by two infinitesimally thin sheets of conductances *G*_1,2_ = *G*_0_ ± *δG*, separated by a dielectric medium of thickness *d* and index of refraction *n*. The propagation-induced differences between fields emitted from the two sheets cancel in (**b**) transmission, but not in (**c**) reflection, leading to vanishing TCD and non-zero RCD.

We consider a single bilayer of oppositely oriented FMs. Each layer is characterized by the index of refraction *n* + *δn*, where the sign of *δn* is opposite for the two layers (Fig. 5a). First, we utilize the transfer matrix approach to reproduce the expressions for the transmission and reflection coefficients of such a bilayer, derived in ref. ^31^. MOKE in the physically relevant limit (*k*_0_*d* ≪ 1 and *δn* ≪ *n*) is given by

2 $${\Theta }_{AFM}=-{k}_{0}nd\frac{2\delta n}{{n}^{2}-1}=\left(-i{k}_{0}nd\right){\Theta }_{FM},$$

where *Θ*_*FM*_ is the Kerr angle of the corresponding bulk ferromagnet, *k*_0_ = *ω*/*c* the wavevector in vacuum, and *n* the average index of refraction of the two layers. The expression for arbitrary layer thickness and *δn* is given in the Supplementary Information.

Eq. (2) showcases the common microscopic origin of the MOKE response of an AFM bilayer and that of the corresponding ferromagnet, but it also reveals a surprising aspect of the link between them. It is natural to assume that a ${{{\mathcal{T}}}}{S}_{1/2}$ - symmetric type-A AFM can exhibit nonzero MOKE only if strong absorption (governed by the imaginary part of *n*) leads to disproportionate sampling of the top layer. However, Eq. (2) shows that this is not correct: the magnitude of *Θ*_*AFM*_ depends on the absolute value of the index, ∣*n*∣, and is nonzero even in the absence of dissipation. The phase of *n* appears in the phase shift between *Θ*_*AFM*_ and *Θ*_*FM*_, which manifests as the difference in the spectrum of RCD between AFM and FM structures, as seen for example in Fig. 3a.

To gain further insight into the physical mechanism behind the alternating FM scenario, we analyze a toy model (Fig. 5b, c) in which each of the two layers is replaced by an infinitesimally thin ferromagnetic sheet of optical conductance *G*_1,2_, where 1, 2 are layer indices. The sheets are separated by a non-magnetic medium of thickness *d* and index of refraction *n*. For illustrative purposes we analyze a single reflection from each sheet.

We consider a circularly polarized plane wave propagating in the positive *z* direction with amplitude *E*_0_. The electric field generates sheet currents,

3 $${K}_{1,2}={G}_{1,2}{E}_{0}{e}^{\mp i{k}_{0}nd/2},$$

where we have taken *z* = 0 as the midpoint between the sheets for convenience. The currents *K*_1,2_ radiate, emitting electric fields,

4 $${E}_{1,2}(z)=\frac{{\mu }_{0}c}{2}{K}_{1,2}{e}^{i{k}_{0}\left|z\pm d/2\right|},$$

where *c* is the speed of light and *μ*_0_ the vacuum permeability.

The total transmitted electric field, given by the sum of *E*_1_(*z*) and *E*_2_(*z*) for *z* > *d*/2, is,

5 $${E}_{T}(z)={\mu }_{0}c{E}_{0}\left(\frac{{G}_{1}+{G}_{2}}{2}\right){e}^{i{k}_{0}z}={\mu }_{0}c{E}_{0}{G}_{0}{e}^{i{k}_{0}z}.$$

As *E*_*T*_(*z*) depends only on the average conductance *G*_0_, the TCD is zero. The toy model shows that this is a consequence of cancellation of propagation factors. The incident wave propagates an additional distance *d* in the medium beyond layer 1 to induce currents layer 2. However, the wave emitted by layer 1 must propagate through the medium to reach a given point *z* > *d*/2. The total propagation factor, *e*^*iknz*^, for waves transmitted through the bilayer is the same, and independent of *d* (Fig. 5b). The sum of fields emitted from the two sheets therefore depends only on the sum of the currents, *G*_1_ + *G*_2_.

In contrast, the propagation factors for fields radiated in the  − *z* direction add, rather than cancel: the incident EM wave travels further to excite current in layer 2 and its radiated field propagates further before reaching an observer at *z* < − *d*/2 (Fig. 5c). The total reflected electric field is given by,

6 $$\begin{array}{rcl}{E}_{R}(z) &=& \frac{{\mu }_{0}c{E}_{0}}{2}{e}^{-i{k}_{0}z}\left({G}_{1}{e}^{-i{k}_{0}nd}+{G}_{2}{e}^{i{k}_{0}nd}\right)\\ & \approx & {\mu }_{0}c{E}_{0}{e}^{-i{k}_{0}z}\left({G}_{0}-\delta Gi{k}_{0}nd\right),\end{array}$$

where we used ∣*k*_0_*nd*∣ ≪ 1 in the expansion of the exponential. The term proportional to *δG* gives rise to *Θ*_*AFM*_.

The toy model for a bilayer identifies the origin of nonzero *Θ*_*AFM*_ as the change in the electric field as it propagates, regardless of whether it is dominated by the real or imaginary component of *n*. We see that absorption does not play a special role in generating MOKE. Further, RCD does not depend on the layer number for even layers (Fig. 4a) because of the exponential nature of propagation: the ratio of fields in two neighboring layers is $\exp (i{k}_{0}nd)$, independent of the number of layers. Thus the effects that yield RCD in two atomic layers and in a semi-infinite AFM crystal are the same. These qualitative features found in the toy model are retained when multiple reflections are included through the transfer matrix approach (Eq. (2), and Fig. 4).

## Discussion

In this paper, we identify alternating FM layers as the origin of MOKE in an A-type antiferromagnet with ${{{\mathcal{T}}}}{S}_{1/2}$ symmetry, therefore providing first experimental confirmation of the mechanism proposed by Dzyaloshinskii and Papamichail^31^. Within this theory, *Θ*_*AFM*_ is entirely determined by the MOKE response of the ferromagnetic layers that are the building blocks of the AFM, allowing us to test it directly by tuning MnBi_2_Te_4_ across the spin-flop transition, thus accessing *Θ*_*AFM*_ and *Θ*_*FM*_ in the same crystal. Based on the quantitative agreement between the measured RCD spectrum in the AFM phase and the theoretical prediction based solely on the FM spectrum, we conclude that MOKE observed at visible and near infrared wavelengths in MnBi_2_Te_4_ arises from alternating FM layers, rather than axion electrodynamics associated with band topology. Axion electrodynamics may dominate the MOKE response at terahertz frequencies below the bulk band gap^25,27,39,40^, where there are no other optical transitions that could overwhelm their contribution, although results in even-layer flakes suggest that the axion contribution in this frequency range is weak^27,39^.

The effect we observe is not limited to MnBi_2_Te_4_, and is generically present in A-type antiferromagnets with spins out of plane. We believe that it has not been recognized experimentally in part because the signal vanishes when averaged over atomic steps, naturally present in as-grown bulk crystals. However, optical experiments in van der Waals materials regularly probe surfaces that are atomically flat across the length scale of a laser beam. We note that some previous sightings of MOKE in van der Waals AFMs with an even number of layers were conditionally attributed to extrinsic magnetization^22,41,42^, given the widespread conviction that MOKE intrinsic to A-type AFMs was forbidden by symmetry. While this explanation is certainly plausible, our findings show that intrinsic MOKE in A-type AFMs is always allowed, and other mechanisms should be considered when *Θ*_*AFM*_ cannot account for the magnitude, or the spectrum, of the observed signal.

Correctly estimating *Θ*_*AFM*_ is therefore essential for interpretation of experimental results. Our work suggests a strategy to do so, both in bulk and thin flakes. In bulk, it is sufficient to combine measured *Θ*_*FM*_ and optical conductivity with the analytical expression for *Θ*_*AFM*_ (Eq. (2)). For thin flakes, it is important to consider the role of the substrate, which may suppress or enhance observed signals^35^; we suggest the transfer matrix approach as a convenient way to do so. We note that ∣*Θ*_*AFM*_∣ is not generically small compared to the noise floor of modern experimental setups: it is suppressed with respect to ∣*Θ*_*FM*_∣ only by a factor of ∣*k*_0_*nd*∣ = ∣2*πnd*/*λ*∣, which is more than an order of magnitude larger than *d*/*λ* suggested by dimensional analysis.

Our findings show that MOKE is a powerful and general tool for investigating ${{{\mathcal{T}}}}{S}_{1/2}$ symmetric AFMs. In this work we used it to image time-reversed domains, as shown for in Fig. 2b. Further, the nonzero *Θ*_*AFM*_ enabled the discovery that a magnetic field can select antiferromagnetic domains (Fig. 2a) in such magnets, challenging the conventional view that antiferromagnets cannot be controlled by external magnetic fields. Uncovering the mechanism behind field control is beyond the scope of this study, but we note that the weakened exchange interaction at the surface is a plausible cause^20,43^. This implies that details of the surface can affect the coercive field, as confirmed by measurements on two flakes exfoliated from the same batch (Fig. S1). However, the magnitude of the RCD at *H* = 0 is a bulk property, independent of local surface conditions. The coercivity and field-switching process will be the subject of future computational^44^ and experimental investigations, enabled by the discovery of the AFM RCD signal. In summary, our results address fundamental questions concerning the origin of MOKE in AFMs, provide a strategy for studying and controlling their domains, and elucidate results on exfoliated van der Waals AFMs.

## Methods

### Complex MOKE: definitions

As discussed in the main text, MOKE is a manifestation of difference in reflectivity between the light of two circular polarizations. The Jones vectors corresponding to the circular polarizations are given by:

M1 $${\widehat{{{{\bf{e}}}}}}_{+}=\frac{1}{\sqrt{2}}\left(\begin{array}{c}1\\ i\end{array}\right),\,({{{\rm{LCP}}}}\,{{{\rm{for}}}}\,{k}_{z} > 0,{{{\rm{RCP}}}}\,{{{\rm{for}}}}\,{k}_{z} < 0)$$

M2 $${\widehat{{{{\bf{e}}}}}}_{-}=\frac{1}{\sqrt{2}}\left(\begin{array}{c}1\\ -i\end{array}\right).\,({{{\rm{RCP}}}}\,{{{\rm{for}}}}\,{k}_{z} > 0,{{{\rm{LCP}}}}\,{{{\rm{for}}}}\,{k}_{z} < 0),$$

capturing the fact that the LCP light becomes RCP upon reflection, and vice-versa. It is convenient to use the ${\widehat{{{{\bf{e}}}}}}_{\pm }$ basis, rather than the LCP and RCP basis, so that reflection from an ideal isotropic mirror is captured by the identity matrix.

Vectors and matrices can be transformed between the ${\widehat{{{{\bf{e}}}}}}_{\pm }$ basis and basis of linear (${\widehat{{{{\bf{e}}}}}}_{x,y}$) polarization using the following transformation matrices:

M3 $${{{{\bf{A}}}}}_{C\to L}=\frac{1}{\sqrt{2}}\left(\begin{array}{cc}1 & 1\\ i & -i\end{array}\right),\,{{{{\bf{A}}}}}_{L\to C}=\frac{1}{\sqrt{2}}\left(\begin{array}{cc}1 & -i\\ 1 & i\end{array}\right).$$

In the ${\widehat{{{{\bf{e}}}}}}_{\pm }$ basis, the reflection matrix corresponding to circular dichroism is given by:

M4 $${r}_{C}=\left(\begin{array}{cc}{r}_{0}+\delta r & 0\\ 0 & {r}_{0}-\delta r\end{array}\right),$$

In the ${\widehat{{{{\bf{e}}}}}}_{x,y}$ basis the reflectivity matrix is then equal to:

M5 $${r}_{L}=\left(\begin{array}{cc}{r}_{0} & {r}_{xy}\\ -{r}_{xy} & {r}_{0}\end{array}\right)=\left(\begin{array}{cc}{r}_{0} & -i\delta r\\ i\delta r & {r}_{0}\end{array}\right).$$

The complex Kerr angle is defined as:

M6 $$\Theta=\frac{{r}_{xy}}{{r}_{0}}=-i\frac{\delta r}{{r}_{0}},$$

and RCD is the imaginary part of *Θ*.

### RCD measurements

The experimental setup is shown in Fig. S4. The cryostat is Quantum Design Opticool, with a 7T magnet. Most measurements were done with a pulsed laser (output of Light Conversion Orpheus optical parametric amplifier, seeded by a Carbide laser, repetition rate 300 kHz, pulse duration  ~ 250 fs), focused to a spot of lateral dimensions of  ~ 4*μ*m. The position dependent measurements (Fig. 2b) were done with a continuous wave laser, with wavelength 532 nm, corresponding to the photon energy of 2.33 eV, which we found to offer good signal to noise throughout the studied field range (Fig. 3a). Spatial resolution is achieved by moving the sample underneath the focused laser spot using Attocube positioners. Regardless of the laser source, the average power of 1*μ*W reached the sample, the incident light was vertically polarized, and chopped using a mechanical chopper.

The incident vertical polarization is rotated by 45 deg using a half wave plate. The next element in the optical path is the photoelastic modulator (PEM) set to a 1/4 wave modulation, therefore modulating light polarization between linear and circular at 50 kHz. The light intensity was measured using a Si photo-diode, through the current input of the Zurich Instruments lock-in amplifier. The intensity was simultaneously demodulated at the chopper and at the PEM frequencies, yielding measured intensities *I*_*c*_ and *I*_*P*_. ${{{\rm{Im}}}}\left(\Theta \right)$ is found through their ratio as (see SI for a derivation):

M7 $${{{\rm{Im}}}}\left(\Theta \right)=\frac{{I}_{P}}{{I}_{c}}\frac{1}{\pi {J}_{1}\left(\pi /2\right)}\approx \frac{{I}_{P}}{{I}_{c}}\frac{1}{1.78073},$$

where *J*_1_ is the Bessel function of first order. Extracting ${{{\rm{Im}}}}\left(\Theta \right)$ through Eq. (M7) has the attractive feature that wavelength-dependent reflection and transmission properties of optical elements, as well as the responsivity of the photodiode, do not influence the measured spectrum, since they cancel out in the ratio $\frac{{I}_{P}}{{I}_{c}}$. Nonetheless, we took into account the wavelength dependence of the photodiode responsivity and the transmission though the beamsplitter to ensure that the power incident on the sample is  ≈ 1*μW* across the spectral range.

Imperfections of optical elements, coupled with the Faraday rotation the cryostat windows and objective, result in a spurious field- and wavelength- dependent additive background in the RCD measurements. We measured the background on a Si/SiO2 substrate, and subtracted it from measurements on MnBi_2_Te_4_ (Fig. S7). Since the spectra in Fig. 3a were obtained through differential measurements, it was not necessary to measure the background contribution for each photon energy.

The error bars in Fig. 3a represent the statistical scatter of the RCD signal within the field range used to extract each value. For the AFM spectrum, the value at *H* = 0 is obtained by averaging the difference between down and up sweeps over the range  ± 0.9 T, and the error bar is the standard deviation of the data points in that range. For the spin-flop spectrum, the value is taken from the difference between two field points (3.4 T and 3.2 T), so the standard deviation is correspondingly larger. Laser power fluctuations do not contribute, since the RCD is normalized by the simultaneously measured reflectivity (measurements were truly simultaneous, in that the same detector voltage is demodulated at two frequencies). The only relevant systematic uncertainty is from the PEM calibration, which here refers to the precision with which the modulation amplitude is known. We use a Hinds PEM-100, whose factory calibration guarantees retardation accuracy within 5% for a narrow monochromatic beam at normal incidence, which is sufficient for our purposes, as this level of uncertainty corresponds to  < 0.1 mrad on the RCD scale in Fig. 3a.

Since our measurements are done with the sample in vacuum, and there are no electrical contacts on the sample, all of the thermalization is happening through the Si/SiO_2_ substrate. This is very inefficient, leading to the known problem of large temperature gradients between the cryostat thermometer and the sample. Independently establishing the sample temperature is therefore difficult, but we use the bulk magnetization measurement as a ‘calibration’ - the agreement between the RCD measurement and the magnetization shows that the sample is at ≈18K.

### Transfer matrix calculations

The reflection and transmission coefficients for the AFM stack are found numerically through transfer matrix calculations, following the method detailed in ref. ^35^. This method offers a computationally convenient way to satisfy boundary conditions on the electric and magnetic fields at each interface, and captures the propagation through a uniform material by a phase factor of $\exp (in{k}_{0}d)$, where *d* is the thickness of the material, *n* the complex index of refraction, and *k*_0_ = *ω*/*c* is the wave vector in vacuum.

We formulate the numerical transfer matrix calculation in the basis of linear polarization. If the dielectric tensor is diagonal in the basis of circular polarization,

M8 $${\varepsilon }_{C}=\left(\begin{array}{cc}{\varepsilon }_{+} & 0\\ 0 & {\varepsilon }_{-},\end{array}\right)$$

in the basis of linear polarization it is given by:

M9 $${\varepsilon }_{L}=\frac{1}{2}\left(\begin{array}{cc}{\varepsilon }_{+}+\varepsilon - & i({\varepsilon }_{-}-\varepsilon+)\\ -i({\varepsilon }_{-}-\varepsilon+) & {\varepsilon }_{+}+\varepsilon -\end{array}\right).$$

This is the form of *ε* we use for transfer matrix calculations, with *ε*_±_ given by Eq. (1). The calculation will return the reflection matrix of the form given by Eq. (M5), with parameters given in Table 1, allowing us to calculate the complex Kerr angle through Eq. (M6), with its imaginary part corresponding to the reflection circular dichroism. A matrix of similar form is found for transmission.

**Table 1:** Parameters of the Lorentz model (Eq. (1)), used for the figures in the main text (Fig. 3b and Fig. 4)

*γ*/*ω*_0_	$f/({\omega }_{0}^{2}\varepsilon )$	*ω*_0_[*eV*]	*δω*[*eV*]	*ε*_*∞*_/*ε*
0.424	4.2	2.05	-0.0708	8.25 + 10.9 *i*

We can use the same formalism to calculate the reflection and transmission from more complex structures. For instance, a calculation of reflection from a small *N* sample suspended in vacuum is unrealistic since samples are necessarily placed on substrates. In Fig. S9 we compare the reflection of a *N* = 8 and *N* = 9 layer sample suspended in vacuum to the same samples on sapphire and diamond substrates, as was done in experiments in ref. ^23^. We find that the *N* = 9 reflection is suppressed more than the *N* = 8 reflection, making the magnitude of the even and odd layer RCD very similar to each other.

### Density functional theory

For our density functional calculations of the frequency-dependent dielectric function for MnBi_2_Te_4_, we employ the Vienna ab-initio software package (VASP)^45^. We use the generalized gradient approximation (GGA) with the Perdew-Burke-Ernzerhof (PBE) functional^46^. The Projector-augmented wave (PAW) method^47^ with the standard VASP pseudopotentials is used, and we take Mn: 3d^6^4s^1^; Bi: 5d^10^6s^2^6p^3^ and Te: 5s^2^5p^4^ electrons as valence. To approximately account for the localized nature of the unpaired d electrons in Mn, we use the DFT+U method^48^ and adopt the rotationally invariant method by Dudarev et al.^49^. We set U=4 eV on the Mn atoms.

Given that MnBi_2_Te_4_ is a layered material, we implement van der Waals corrections, using the DFT-D3 method of Grimme et al.^50^. We relax the bulk, primitive rhombohedral cell of MnBi_2_Te_4_ using a Gamma-centered mesh of 13 × 13 × 5 to sample the Brillouin zone and a kinetic energy cutoff of 600 eV for our plane-wave basis set. For the structural relaxation, we neglect spin–orbit coupling (SOC), and enforce ferromagnetic order of the Mn ions using spin-polarized collinear calculations. We use a Gaussian smearing for the partial occupancies with a smearing width of 0.01 eV.

To calculate the frequency-dependent dielectric tensor within the independent particle approximation (that is, neglecting local field effects), we use the method developed by Gajdoš et al.^51^. For these calculations we include SOC self-consistently and enforce ferromagnetic ordering with the spin axis perpendicular to the Mn layers. Having first obtained the ground-state electronic density and corresponding Kohn-Sham states in a self-consistent DFT calculation, the imaginary part of *ε* is calculated with these wavefunctions using the following equation:

M10 $${\varepsilon }_{\alpha \beta }^{(2)}(\omega )=	 \frac{4{\pi }^{2}{e}^{2}}{\Omega }{lim}_{q\to 0}\frac{1}{{q}^{2}}{\sum }_{c,v,{{{\bf{k}}}}}2{w}_{{{{\bf{k}}}}}\delta ({\epsilon }_{c{{{\bf{k}}}}}-{\epsilon }_{v{{{\bf{k}}}}}-\omega ) \\ 	 \,\times \langle {u}_{c{{{\bf{k}}}}+{{{{\bf{e}}}}}_{\alpha }q}| {u}_{v{{{\bf{k}}}}}\rangle {\langle {u}_{c{{{\bf{k}}}}+{{{{\bf{e}}}}}_{\beta }q}| {u}_{v{{{\bf{k}}}}}\rangle }^{*},$$

Here, *w*_*k*_ is the weight of point *k* in the discrete summation over the Brillouin zone, *Ω* is the volume of the unit cell, and the indices *c* and *v* denote conduction and valence bands respectively, with *ϵ*_*c*_ and *ϵ*_*v*_ being their respective energies at the wavevector k. e_*α*_ is the unit vector in Cartesian direction *α*. To get the real part of the dielectric function we use the Kramers-Kronig transformation

M11 $${\varepsilon }_{\alpha \beta }^{(1)}(\omega )=1+\frac{2}{\pi }{{{\mathcal{P}}}}{\int }_{0}^{\infty }\frac{{\omega }^{{\prime} }{\varepsilon }_{\alpha \beta }^{(2)}({\omega }^{{\prime} })}{\omega {{\prime} }^{2}-{\omega }^{2}+i\eta }d{\omega }^{{\prime} },$$

Where ${{{\mathcal{P}}}}$ is the principal value and *η* is a Lorentzian broadening. For both the self-consistent DFT calculation to obtain converged wave functions and evaluation of the dielectric function, we use a 17 × 17 × 5 Gamma-centered mesh, and we use 540 bands to ensure that we have sufficient unoccupied bands for convergence of the summation. We select a Lorentzian broadening of 0.1 eV.

### Crystal growth, sample preparation, and thickness measurements

MnBi_2_Te_4_bulk crystals were grown using a Bi-Te flux, following the previously reported recipe in ref. ^32^. The magnetization data in fields up to 12 T were collected using the AC option of a Quantum Design physical property measurement system.

In this work, we study thin bulk samples (infinite layer) with a thickness of 136 nm, corresponding to approximately 100 septuple layers. These samples are similar to bulk crystals and are not air-sensitive, in contrast to atomically thin flakes which can degrade in air over time^19,21^. Nevertheless, care has been taken to minimize sample exposure to air before measurements.

To obtain thin bulk samples with atomically flat surfaces suitable for RCD studies, we used Scotch tape exfoliation of bulk crystals onto silicon wafers covered with 285 nm SiO_2_ (we used 3M Magic Scotch Tape). The silicon wafers were pre-treated with RF O_2_ plasma (duration: 10 min; power: 80 W; O_2_ gas flow: 10 SCCM) before exfoliation to increase adhesion of crystals and overall yield. The exfoliation process was performed entirely inside a glovebox filled with inert argon gas, maintaining O_2_ and H_2_O levels below 0.1 ppm.

Once thin bulk samples of suitable dimensions were identified using an optical microscope, they were transported for loading into the low-temperature cryostat with optical access using a sealed container filled with argon. Care was taken to minimize air exposure during cryostat loading; we estimate that the sample was exposed to air for a maximum of 5 min before measurements. Based on the careful studies of oxidation on MnBi_2_Te_4_^52^, it is possible that the surface layer oxidized. However, the oxidation is self-limiting to the surface atomic layer^52^, and therefore cannot dominate our optical measurements with 30 nm penetration depth. The height of the studied thin bulk sample was measured after RCD measurements were completed using atomic force microscopy (Asylum Research Cypher S, tapping mode) in air.

## Supplementary information

Supplementary Information

Peer Review File

## Data Availability

The datasets generated and analyzed during the study of “Magneto-optical Kerr effect in an A-type antiferromagnet" are available in the ISTA REx repository with https://doi.org/10.15479/AT-ISTA-21422.

## References

[1] Gong, C. et al. Discovery of intrinsic ferromagnetism in two-dimensional van der waals crystals. *Nature* **546**, 265 (2017). 10.1038/nature22060 28445468

[2] Huang, B. et al. Layer-dependent ferromagnetism in a van der waals crystal down to the monolayer limit. *Nature* **546**, 270 (2017). 10.1038/nature22391 28593970

[3] Mak, K. F., Shan, J. & Ralph, D. C. Probing and controlling magnetic states in 2D layered magnetic materials. *Nat. Rev. Phys.* **1**, 646 (2019).

[4] Fei, Z. et al. Two-dimensional itinerant ferromagnetism in atomically thin Fe3GeTe2. *Nat. Mater.* **17**, 778 (2018). 10.1038/s41563-018-0149-7 30104669

[5] Gibertini, M., Koperski, M., Morpurgo, A. F. & Novoselov, K. S. Magnetic 2D materials and heterostructures. *Nat. Nanotechnol.* **14**, 408 (2019). 10.1038/s41565-019-0438-6 31065072

[6] Huang, B. et al. Emergent phenomena and proximity effects in two-dimensional magnets and heterostructures. *Nat. Mater.* **19**, 1276 (2020). 10.1038/s41563-020-0791-8 32948831

[7] Zhao, W. et al. Emergence of ferromagnetism at the onset of moiré Kondo breakdown. *Nat. Phys.* **20**, 1772 (2024).

[8] Sun, Z. et al. Resolving and routing magnetic polymorphs in a 2D layered antiferromagnet. *Nat. Mater.* **24**, 226 (2025). 10.1038/s41563-024-02074-w 39805959

[9] Park, H. et al. Ferromagnetism and topology of the higher flat band in a fractional Chern insulator. *Nat. Phys.* **21**, 549–555 (2025).

[10] Baltz, V. et al. Antiferromagnetic spintronics. *Rev. Mod. Phys.* **90**, 015005 (2018).

[11] Němec, P., Fiebig, M., Kampfrath, T. & Kimel, A. V. Antiferromagnetic opto-spintronics. *Nat. Phys.* **14**, 229 (2018).

[12] Han, J. et al. Coherent antiferromagnetic spintronics. *Nat. Mater.* **22**, 684 (2023). 10.1038/s41563-023-01492-6 36941390

[13] Chen, H. et al. Emerging Antiferromagnets for Spintronics. *Adv. Mater.* **36**, 2310379 (2024). 10.1002/adma.202310379 38183310

[14] Šmejkal, L., MacDonald, A. H., Sinova, J., Nakatsuji, S. & Jungwirth, T. Anomalous Hall antiferromagnets. *Nat. Rev. Mater.* **7**, 482 (2022).

[15] S. F., Weber, surface magnetization in antiferromagnets: classification, example materials, and relation to magnetoelectric responses. *Phys. Rev.* X**14**, 10.1103/PhysRevX.14.021033 (2024).

[16] Krichevtsov, B. B., Pavlov, V. V., Pisarev, R. V. & Gridnev, V. N. Spontaneous non-reciprocal reflection of light from antiferromagnetic Cr2O3. *J. Phys. Condens. Matter* **5**, 8233 (1993).

[17] Higo, T. et al. Large magneto-optical Kerr effect and imaging of magnetic octupole domains in an antiferromagnetic metal. *Nat. Photonics* **12**, 73 (2018). 10.1038/s41566-017-0086-z PMC5997294 29910828

[18] Wu, M., Isshiki, H., Chen, T., Higo, T., Nakatsuji, S. & Otani, Y. Magneto-optical Kerr effect in a non-collinear antiferromagnet Mn3Ge. *Appl. Phys. Lett.* **116**, 132408 (2020).

[19] Otrokov, M. M. et al. Prediction and observation of an antiferromagnetic topological insulator. *Nature* **576**, 416 (2019). 10.1038/s41586-019-1840-9 31853084

[20] Sass, P. M. et al. Robust *A*-type order and spin-flop transition on the surface of the antiferromagnetic topological insulator MnBi_2_Te_4_. *Phys. Rev. Lett.* **125**, 037201 (2020). 10.1103/PhysRevLett.125.037201 32745385

[21] Ovchinnikov, D. et al. Intertwined topological and magnetic orders in atomically thin chern insulator MnBi_2_Te_4_. *Nano Lett.* **21**, 2544 (2021). 10.1021/acs.nanolett.0c05117 33710884

[22] Yang, S. Odd-even layer-number effect and layer-dependent magnetic phase diagrams in MnBi_2_Te_4_. *Phys. Rev. X* **11**, 011003 (2021).

[23] Qiu, J.-X. et al. Axion optical induction of antiferromagnetic order. *Nat. Mater.* **22**, 583 (2023). 10.1038/s41563-023-01493-5 36894774

[24] Bartram, F. M. et al. Real-time observation of magnetization and magnon dynamics in a two-dimensional topological antiferromagnet MnBi_2_Te_4_. *Sci. Bull.* **68**, 2734 (2023). 10.1016/j.scib.2023.10.003 37863774

[25] Ahn, J., Xu, S.-Y. & Vishwanath, A. Theory of optical axion electrodynamics and application to the Kerr effect in topological antiferromagnets. *Nat. Commun.* **13**, 7615 (2022). 10.1038/s41467-022-35248-8 PMC9734152 36494356

[26] Essin, A. M., Moore, J. E. & Vanderbilt, D. Magnetoelectric polarizability and axion electrodynamics in crystalline insulators. *Phys. Rev. Lett.* **102**, 146805 (2009). 10.1103/PhysRevLett.102.146805 19392469

[27] Lei, C. & MacDonald, A. H. Kerr, Faraday, and magnetoelectric effects in MnBi_2_Te_4_ thin films. *Phys. Rev. B* **108**, 125424 (2023).

[28] Wilczek, F. Two applications of axion electrodynamics. *Phys. Rev. Lett.* **58**, 1799 (1987). 10.1103/PhysRevLett.58.1799 10034541

[29] Hornreich, R. M. & Shtrikman, S. Theory of gyrotropic birefringence. *Phys. Rev.* **171**, 1065 (1968).

[30] Orenstein, J. Optical nonreciprocity in magnetic structures related to high-T_c_ superconductors. *Phys. Rev. Lett.* **107**, 067002 (2011). 10.1103/PhysRevLett.107.067002 21902360

[31] Dzyaloshinskii, I. & Papamichail, E. V. Nonreciprocal optical rotation in antiferromagnets. *Phys. Rev. Lett.* **75**, 3004 (1995). 10.1103/PhysRevLett.75.3004 10059464

[32] Yan, J.-Q. et al. Crystal growth and magnetic structure of MnBi_2_Te_4_. *Phys. Rev. Mater.* **3**, 064202 (2019).

[33] Köpf, M., Ebad-Allah, J., Lee, S. H., Mao, Z. Q. & Kuntscher, C. A. Influence of magnetic ordering on the optical response of the antiferromagnetic topological insulator MnBi_2_Te_4_. *Phys. Rev. B* **102**, 165139 (2020).

[34] Zak, J., Moog, E. R., Liu, C. & Bader, S. D. Universal approach to magneto-optics. *J. Magn. Magn. Mater.* **89**, 107 (1990).

[35] Hendriks, F. & Guimarães, M. H. D. Enhancing magneto-optic effects in two-dimensional magnets by thin-film interference. *AIP Adv.* **11**, 035132 (2021).

[36] Dzyaloshinskii, I. E. Space and time parity violation in anyonic and chiral systems. *Phys. Lett. A* **155**, 62 (1991).

[37] Canright, G. S. & Rojo, A. G. Ellipsometry and broken time-reversal symmetry in the high-temperature superconductors. *Phys. Rev. B* **46**, 14078 (1992). 10.1103/physrevb.46.14078 10003478

[38] Armitage, N. P. Constraints on Jones transmission matrices from time-reversal invariance and discrete spatial symmetries. *Phys. Rev. B* **90**, 035135 (2014).

[39] X., Han, A.-H., Chen, M., Brahlek, and L., Wu, Quantized magneto-terahertz effects in the antiferromagnetic topological insulator MnBi_2_Te_4_ Thin Films(2025), https://arxiv.org/abs/2503.13651.

[40] Wu, L. et al. Quantized Faraday and Kerr rotation and axion electrodynamics of a 3D topological insulator. *Science* **354**, 1124 (2016). 10.1126/science.aaf5541 27934759

[41] Huang, B. et al. Electrical control of 2D magnetism in bilayer cri3. *Nat. Nanotechnol.* **13**, 544 (2018). 10.1038/s41565-018-0121-3 29686292

[42] Zhang, X.-X. et al. Gate-tunable spin waves in antiferromagnetic atomic bilayers. *Nat. Mater.* **19**, 838 (2020). 10.1038/s41563-020-0713-9 32572203

[43] Chong, S. K. et al. Intrinsic exchange biased anomalous Hall effect in an uncompensated antiferromagnet MnBi2Te4. *Nat. Commun.* **15**, 2881 (2024). 10.1038/s41467-024-46689-8 PMC10991375 38570519

[44] S. F., Weber and V., Sunko, Deterministic domain selection of antiferromagnets via magnetic fields (2026), https://arxiv.org/abs/2601.06646.

[45] Kresse, G. & Furthmüller, J. Efficient iterative schemes for ab initio total-energy calculations using a plane-wave basis set. *Phys. Rev. B* **54**, 11169 (1996). 10.1103/physrevb.54.11169 9984901

[46] Perdew, J. P., Burke, K. & Ernzerhof, M. Generalized gradient approximation made simple. *Phys. Rev. Lett.* **77**, 3865 (1996). 10.1103/PhysRevLett.77.3865 10062328

[47] Blöchl, P. E. Projector augmented-wave method. *Phys. Rev. B* **50**, 17953 (1994). 10.1103/physrevb.50.17953 9976227

[48] Anisimov, V. I., Aryasetiawan, F. & Lichtenstein, A. I. First-principles calculations of the electronic structure and spectra of strongly correlated systems: the LDA+ U method. *J. Phys. Condens. Matter* **9**, 767 (1997).

[49] Dudarev, S. L., Botton, G. A., Savrasov, S. Y., Humphreys, C. J. & Sutton, A. P. Electron-energy-loss spectra and the structural stability of nickel oxide: an LSDA+U study. *Phys. Rev. B* **57**, 1505 (1998).

[50] Grimme, S., Antony, J., Ehrlich, S. & Krieg, H. A consistent and accurate ab initio parametrization of density functional dispersion correction (DFT-D) for the 94 elements H-Pu. *J. Chem. Phys.* **132**, 154104 (2010). 10.1063/1.3382344 20423165

[51] Gajdoš, M., Hummer, K., Kresse, G., Furthmüller, J. & Bechstedt, F. Linear optical properties in the projector-augmented wave methodology. *Phys. Rev. B* **73**, 045112 (2006).

[52] Mazza, A. R. et al. Surface-driven evolution of the anomalous hall effect in magnetic topological insulator MnBi2Te4 thin films, *Adv. Funct. Mater*. **32**, 2202234. 10.1002/adfm.202202234 (2022).

