# Supplementary material for: Magneto-optical Kerr effect in an A-type antiferromagnet
Source: Nat Commun. 2026 May 12;17:7364. doi: 10.1038/s41467-026-72577-4 (PMC13402694; doi:10.1038/s41467-026-72577-4)
Supplement: Supplementary file 1 — Supplementary Information [file 41467_2026_72577_MOESM1_ESM.pdf]

SUPPLEMENTARY INFORMATION FOR 'MAGNETO-OPTICAL KERR EFFECT IN AN A-TYPE ANTIFERROMAGNET'

The analytical solutions

Bulk ferromagnet

The reflectivity matrix of a ferromagnet in the $\hat{\mathbf{e}}_\pm$ basis is:

$$r_C^{FM} = \begin{pmatrix} r_0 + \delta r & 0 \\ 0 & r_0 - \delta r \end{pmatrix} = \begin{pmatrix} \frac{1-n-\delta n}{1+n+\delta n} & 0 \\ 0 & \frac{1-n+\delta n}{1+n-\delta n}, \end{pmatrix}. \tag{S1}$$

where $n_{1,2} = n \pm \delta n$ are the indices of refraction for $\hat{\mathbf{e}}_+$ and $\hat{\mathbf{e}}_-$, that is for LCP and RCP incident light, respectively. This leads to:

$$\delta r = \frac{1}{2}\left(\frac{1-n-\delta n}{1+n+\delta n} - \frac{1-n+\delta n}{1+n-\delta n}\right) = \frac{-2\delta n}{-\delta n^2 + n^2 + 2n + 1} \tag{S2}$$

and

$$r_0 = \frac{\delta n^2 - n^2 + 1}{-\delta n^2 + n^2 + 2n + 1}, \tag{S3}$$

yielding the complex Kerr angle (Eq. M6):

$$\Theta_{FM} = (-i)\left(-\frac{2\delta n}{\delta n^2 - n^2 + 1}\right) \approx \frac{-2i\delta n}{n^2 - 1}. \tag{S4}$$

Antiferromagnetic bilayer

We use the transfer matrix method to analytically solve for the reflection and transmission of an antiferromagnetic bilayer suspended in vacuum (Fig. 5a). The indices of refraction for $\hat{\mathbf{e}}_+$ polarization of the two layers $n_{1,2} = n \pm \delta n$, and $n_{1,2} = n \mp \delta n$ for $\hat{\mathbf{e}}_-$. The thickness of the individual layers is d, and $k_0 = \omega/c$, were ω and c are the frequency and speed of light.

We formulate the calculation in the basis of circular polarization, so that the problem is diagonal. We find that the transmission circular dichroism vanishes identically. In contrast, for reflectivity we find:

$$\frac{\delta r}{r_0} = \frac{2i\,\delta n\, n\,(\cos(2dk_0 n) - \cos(2dk_0\delta n))}{n(-\delta n^2 + n^2 - 1)\sin(2dk_0 n) + \delta n(-\delta n^2 + n^2 + 1)\sin(2dk_0\delta n)} \tag{S5}$$

The complex MOKE angle can be found using Eq M6. In the physically relevant limit of $dk_0 \ll 1$, $\delta n \ll n$, this expression reduces to

$$\Theta_{AFM} = (-i)\frac{\delta r}{r_0} \approx -\frac{2\delta n}{n^2-1}(dk_0 n) = -\frac{2i\delta n}{n^2-1}(-idk_0 n) = \Theta_{FM}(-idk_0 n) \qquad \text{(S6)}$$

where Θ_{FM} stands for the complex Kerr angle of a bulk ferromagnet described by the $n \pm \delta n$ for $\hat{\mathbf{e}}_+$.

Photoelastic modulator and the RCD measurement

The time-dependent effect of the photoelastic modulator (PEM) can be captured in the basis of linear polarization with the following Jones matrix:

$$J_{PEM} = \begin{pmatrix} e^{-\frac{1}{4}i\pi \sin(t\omega_P)} & 0 \\ 0 & e^{\frac{1}{4}i\pi \sin(t\omega_P)} \end{pmatrix}, \qquad \text{(S7)}$$

where ω_P is the angular frequency of polarization modulation. The modulated light is reflected from the sample, and focused onto a photodiode. The modulation of total reflectivity that is synchronous with the polarization modulation is proportional to the imaginary part of Θ, which can be seen from a Jones matrix calculation, outlined below.

The reflected electric field is given by,

$$\begin{pmatrix} E_x \\ E_y \end{pmatrix} = r_L \times J_{PEM} \times \frac{1}{\sqrt{2}}\begin{pmatrix} 1 \\ 1 \end{pmatrix}, \qquad \text{(S8)}$$

where r_L is the sample reflectivity expressed in the linear basis. The measured quantity is intensity,

$$I = |E|^2 \left[\frac{1}{2}\left(1 + \mathrm{sgn}\left(\sin\left(\omega_c t\right)\right)\right)\right], \qquad \text{(S9)}$$

where the square wave term in the square brackets accounts for the modulation by the mechanical chopper at frequency ω_c, and $|E|$ is the absolute value of the reflected electric field. Keeping only the first term in the Fourier transform of the square wave, we have:

$$I = |E|^2 \left[\frac{1}{2} + \frac{2}{\pi}\sin\left(\omega_c t\right)\right]. \qquad \text{(S10)}$$

To calculate $|E|^2$, we use Eqs. M5, S7 and S8, and find:

$$\frac{|E|^2}{|r_0|^2} = 1 + \left|\frac{r_{xy}}{r_0}\right|^2 + 2\,\mathrm{Im}\left(\frac{r_{xy}}{r_0}\right)\sin\left(\frac{1}{2}\pi\sin(\omega_P t)\right) \approx 1 + 4J_1\left(\frac{\pi}{2}\right)\mathrm{Im}(\Theta)\sin(\omega_P t)\,, \quad \text{(S11)}$$

where $J_1(\pi/2)$ is the Bessel function of the first kind. In the last step we used the Jacobi-Anger expansion, and omitted the quadratic term in r_{xy}/r_0. The key point is that this signal contains information on $\mathrm{Im}(\Theta)$.

The intensity can be expressed as:

$$\frac{I}{|r_0|^2} = \left(1 + 4J_1\left(\frac{\pi}{2}\right)\mathrm{Im}(\Theta)\sin(\omega_P t)\right)\left[\frac{1}{2} + \frac{2}{\pi}\sin(\omega_c t)\right] \quad \text{(S12)}$$

$$= \frac{1}{2} + \frac{2}{\pi}\sin(\omega_c t) + 2J_1\left(\frac{\pi}{2}\right)\mathrm{Im}(\Theta)\sin(\omega_P t) + \frac{8}{\pi}J_1\left(\frac{\pi}{2}\right)\mathrm{Im}(\Theta)\sin(\omega_P t)\sin(\omega_c t). \quad \text{(S13)}$$

We simultaneously demodulate the measured intensity at ω_c and ω_P using a lock-in amplifier, yielding currents I_c and I_P, respectively. We find:

$$\frac{I_P}{I_c} = \pi J_1\left(\frac{\pi}{2}\right)\mathrm{Im}(\Theta)\,, \quad \text{(S14)}$$

or, equivalently,

$$\mathrm{Im}(\Theta) = \frac{I_P}{I_c}\frac{1}{\pi J_1(\pi/2)} \approx \frac{I_P}{I_c}\frac{1}{1.78073}. \quad \text{(S15)}$$

EFFECT OF SURFACE QUALITY ON COERCIVE FIELD AND ZERO-FIELD RCD

The coercive field in our measurements varies between nominally identical flakes exfoliated from the same $MnBi_2Te_4$ crystal, whereas the magnitude of the reflection circular dichroism (RCD) at $H = 0$ remains constant. This observation is illustrated in Fig. S1, in which we compare two flakes placed on the same substrate and measured in the same cooldown. While the zero-field RCD values are identical within experimental error, the coercive fields and the shapes of the hysteresis differ.

We attribute this difference in coercivity to variations in local surface conditions, such as strain introduced during exfoliation or subtle differences in surface oxidation. We know that the Hamiltonian parameters of the surface spin (exchange coupling, anisotropy) are different from those of the bulk, and as we point in the main text this difference is crucial for magnetic-field tuning of AFM domains. The difference between the Hamiltonian properties at the surface and the bulk can of course be influenced by details of the surface, leading

to the different coercivity. However, surface oxidation in $MnBi_2Te_4$ is self-limiting and confined to the topmost atomic layer [52], and the optical penetration depth far exceeds this layer thickness. Consequently, while the surface environment can affect the switching field of the antiferromagnetic domains, it cannot account for the robust and reproducible RCD magnitude at $H = 0$.

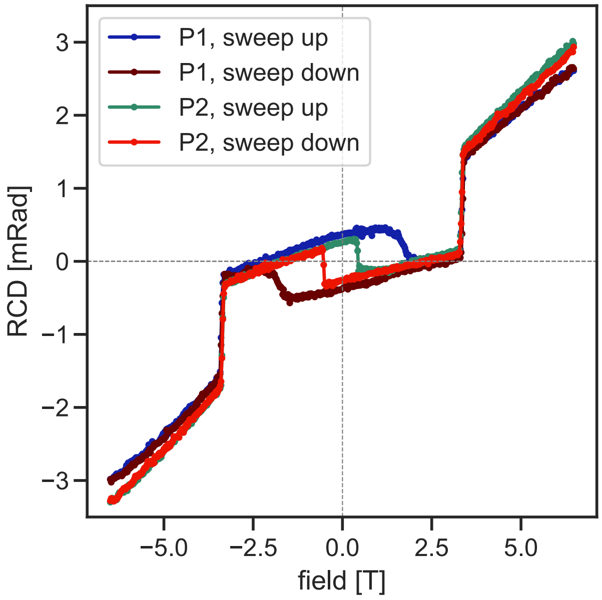

Figure S1. Flake-to-flake variation. RCD as a function of field for two different bulk $MnBi_2Te_4$ flakes, measured at a wavelength of 570 nm. While the coercive field varies between flakes, the zero-field RCD magnitude is identical within experimental error. This demonstrates that the coercive field is sensitive to surface-specific details, whereas the zero-field RCD is a robust property of the bulk antiferromagnetic state.

To further test whether surface modifications could mimic our results, we simulated RCD spectra for three scenarios: (a) pristine A-type AFM with domain reversal; (b) A-type AFM with domain reversal, with a non-magnetic surface layer; (c) AFM with a ferromagnetic surface layer that switches independently of the bulk. Only scenarios (a,b), in which the AFM domain switches, reproduce the experimental finding that the two zero-field RCD magnitudes are equal in magnitude and opposite in sign (Fig. S7). In particular, switching only a surface ferromagnetic layer yields asymmetric zero-field spectra, inconsistent with the data (Fig. S2).

These findings establish that the reproducible zero-field RCD signal arises from reversal

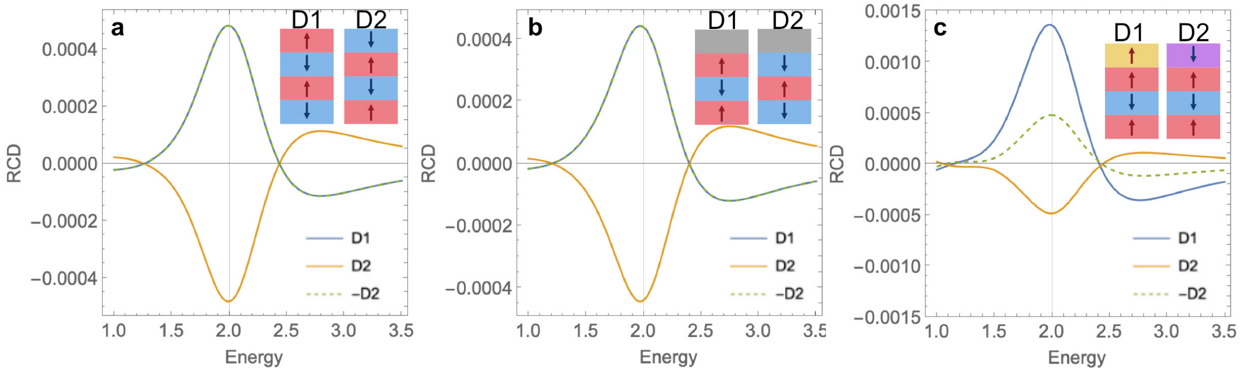

Figure S2. Effect of surface modifications on zero-field RCD. We show simulated RCD spectra for three scenarios: (a) pristine A-type AFM with domain reversal; (b) A-type AFM with domain reversal and a non-magnetic surface layer; and (c) A-type AFM with a ferromagnetic surface layer that switches independently of the bulk. Each panel shows RCD as a function of photon energy for the two AFM domain configurations sketched in the corner. Only scenarios (a) and (b), in which the AFM domain switches, reproduce the experimental observation that the two zero-field RCD spectra are equal in magnitude and opposite in sign. Switching only a surface ferromagnetic layer yields asymmetric zero-field spectra, inconsistent with the experiment.

of bulk AFM domains. While the coercive field is sensitive to details of the surface, this does not influence the magnitude of the RCD at $H = 0$, which is a robust signature of the underlying bulk antiferromagnetic order.

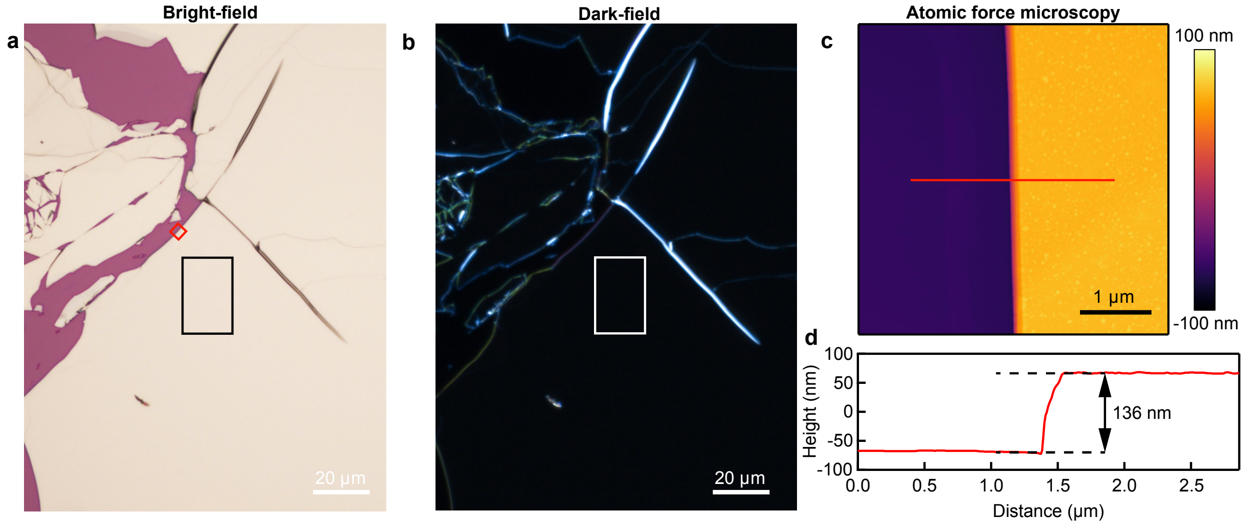

Figure S3. Optical and atomic force microscopy characterization of thin bulk $MnBi_2Te_4$. (a) Optical micrograph (bright field) of the region of interest. The black rectangle outlines the area where RCD sweeps and maps were taken. The red rectangle shows the area where the height was measured using atomic force microscopy. (b) Optical micrograph (dark field) of the same region with a white rectangle demonstrating the area where RCD was measured. (c) Atomic force microscopy image of the edge of the $MnBi_2Te_4$ thin bulk flake used for optical measurements (outlined in the red rectangle in panel (a)). (d) The height profile measured along the red line in (c) which measures the height of the thin bulk to be 136 nm.

γ/ω_0	$f/(\omega_0^2\varepsilon)$	$\omega_0[eV]$	$\delta\omega[eV]$	$\varepsilon_\infty/\varepsilon$
0.424	4.2	2.05	-0.0708	8.25 + 10.9 i

Table S1. Parameters of the Lorentz model (Eq. 1), used for the figures in the main text (Fig. 3b and Fig. 4).

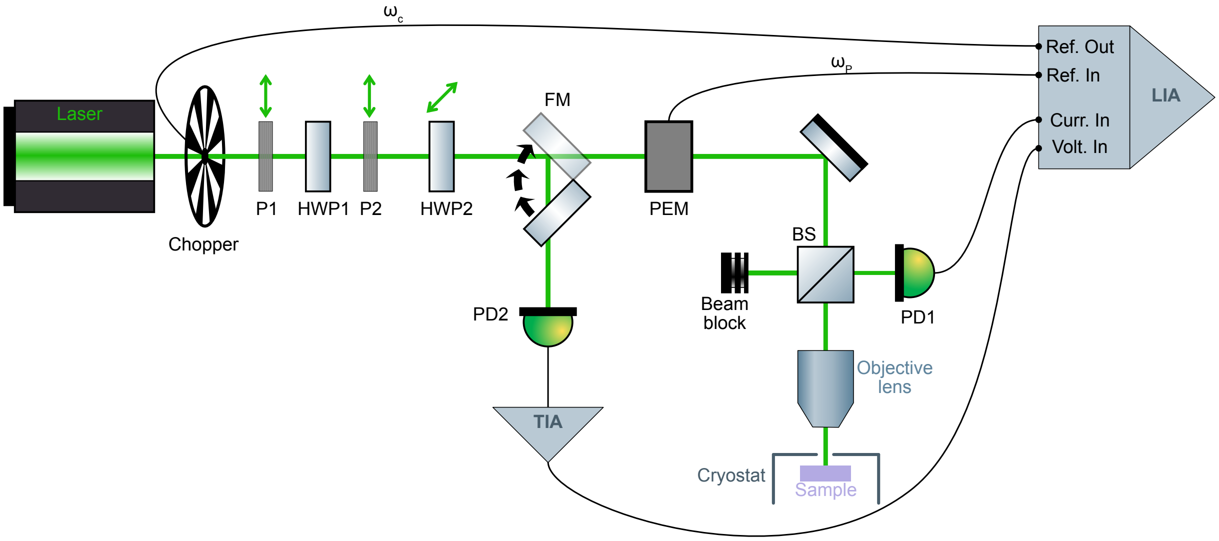

Figure S4. Optical setup used for reflection circular dichroism (RCD) measurements. The laser beam is modulated by a mechanical chopper at frequency ω_c, and directed toward the sample via a series of optics which control light intensity and polarization. The first half-wave plate (HWP1), placed between vertical polarizers P1 and P2, is used to control the intensity. HWP2 sets the polarization angle to 45° with respect to the modulation axis of the photoelastic modulator (PEM), which modulates the helicity of the beam at $\omega_P = 50\,\text{kHz}$. A flip mirror (FM) directs the light towards photodiode PD2 before the measurement at each wavelength, to ensure that the same intensity reaches the sample at each wavelength. The beam is focused onto the sample by a 10x objective (NA=0.25) that is outside of the cryostat. The reflected light is split by a beam splitter (BS), and focused onto photdiode PD1. The signals are read through the Zurich Instruments MFLI lock-in amplifier with two demodulators, at frequencies ω_c and ω_P. The current from PD1 is read through the low-noise current input of the LIA, while the current from PD2 is transformed into voltage by a transimpedance amplifier (TIA), and read through the voltage input of the same LIA.

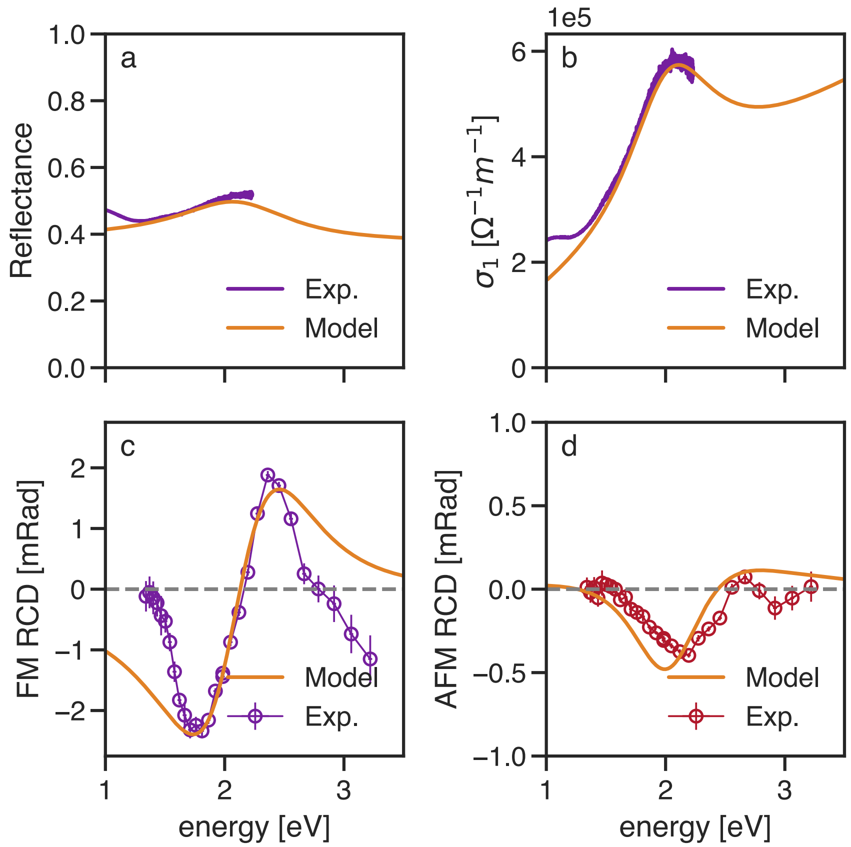

Figure S5. Comparison of the experimental data to the Lorentz model. (a) Reflectance (R), (b) the real part of conductivity (σ_1) and RCD of a (c) ferromagnet and (d) antiferromagnet calculated using the dielectric function described by the Lorentz model (Eq. 1, parameters listed in Table S1), compared to experimental values. The experimental data for R and σ_1 were published in Ref. [33], while RCD is measured as a part of this work. The FM RCD curve from the model is multiplied by 0.4, since the measured jump in RCD corresponds to 0.4 of the saturation magnetization value at this temperature. The AFM RCD is calculated using the transfer matrix approach, assuming that each layer shares the dielectric properties of the bulk FM, with alternating spin orientation.

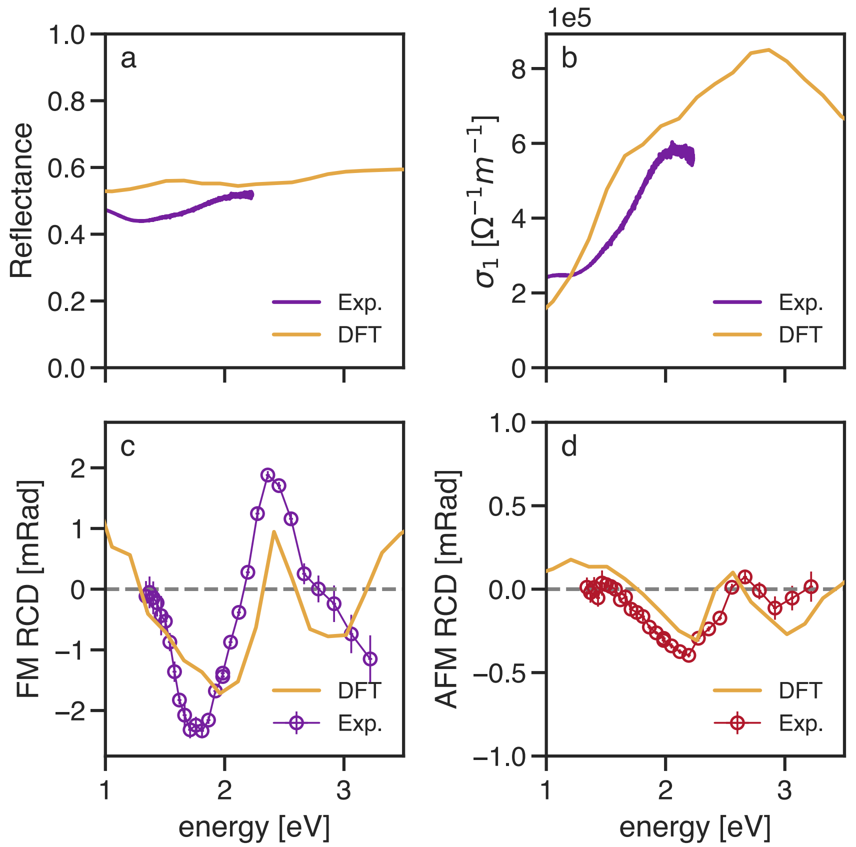

Figure S6. Comparison of the experimental data to the quantities calculated from the DFT calculation. (a) Reflectance (R), (b) the real part of conductivity (σ_1) and RCD of (c) a ferromagnet and (d) an antiferromagnet calculated using density functional theory, compared to experimental values. The experimental data for R and σ_1 were published in Ref. [33], while RCD is measured as a part of this work. The DFT calculation is done for the polarized FM ground state, and the ordered moment is found to be equal to 4.548 μ_B/Mn. To directly compare with the experimental data we introduce the following scaling: the DFT FM RCD is multiplied by 1.2/4.548, reflecting the magnitude of the magnetization change at the spin-flop transition at 15 K, while the DFT AFM RCD curve is multiplied by 3/4.548, reflecting the fact that the ordered moment at 15 K is 3 μ_B/Mn. The AFM RCD is calculated using the transfer matrix approach, assuming that each layer shares the dielectric properties of the bulk FM, with alternating spin orientation. The DFT data in all four panels were rigidly shifted by 300 meV.

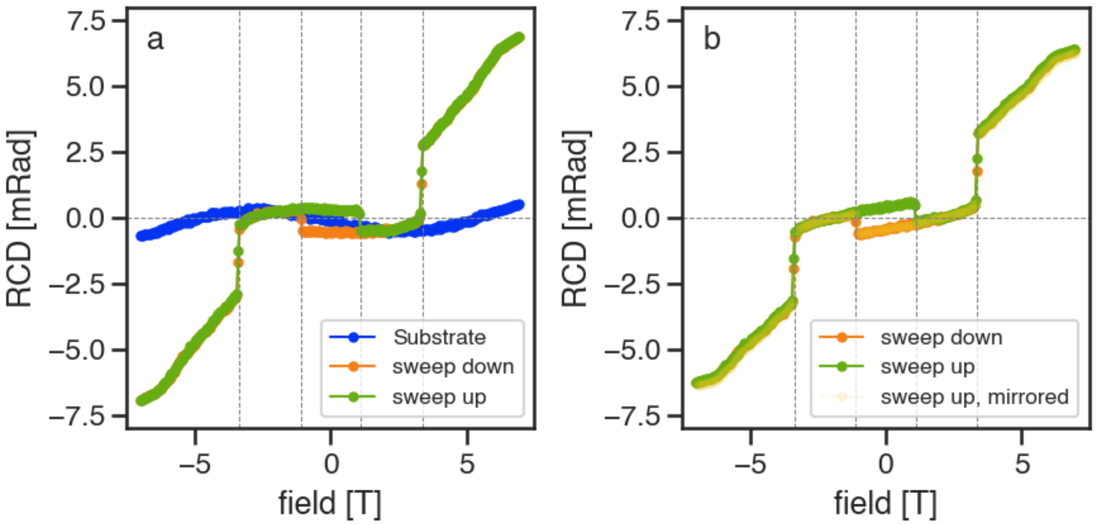

Figure S7. RCD background subtraction. (a) RCD measured on a non-magnetic Si/SiO_2 substrate, as well as RCD measured on $MnBi_2Te_4$ on upward and downward field sweeps. (b) The measured signal with the background subtracted, also shown in Fig. 2a. We include the mirrored version of the sweep up curve, which perfectly aligns with the sweep down curve. This indicates that the two states at $H = 0$ are time-reversed versions of each other, and is inconsistent with the hypothesis that only a top surface ferromagnetic spin is reoriented by the magnetic field.

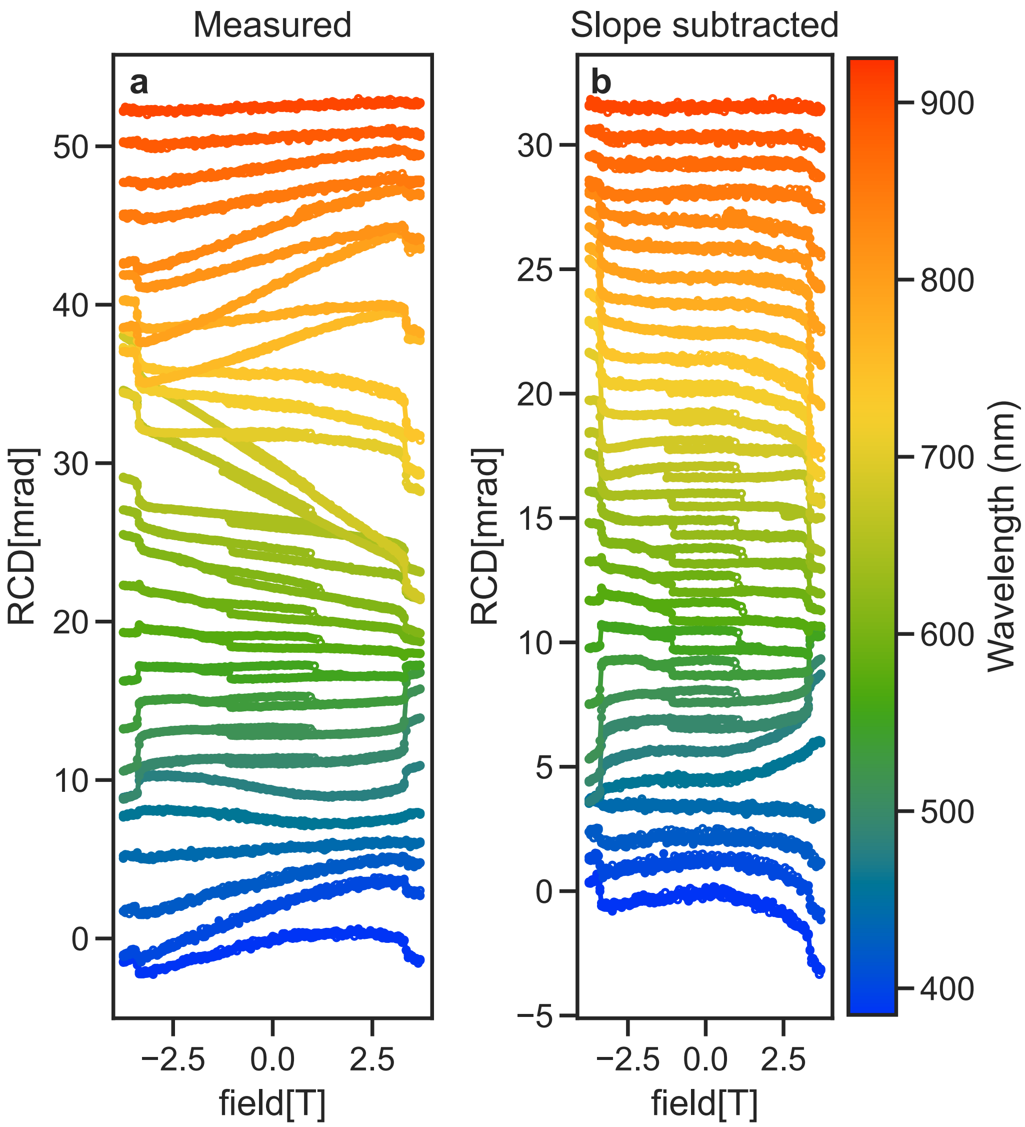

Figure S8. RCD vs field for the range of wavelengths used in Fig. 3a. (a) The measured RCD vs magnetic field, measured at a range of wavelengths. (b) Same as (a), but with the slope at $H = 0$ subtracted for clarity. Setup artifacts contribute a field- and wavelength- dependent background to these measurements, however they do not impact the extracted values of RCD at $H = 0$ or across the spin-flop transition, because both are obtained through differential measurements, that cancel the background effects (see Fig. 3a, and the corresponding caption). Curves are offset for clarity.

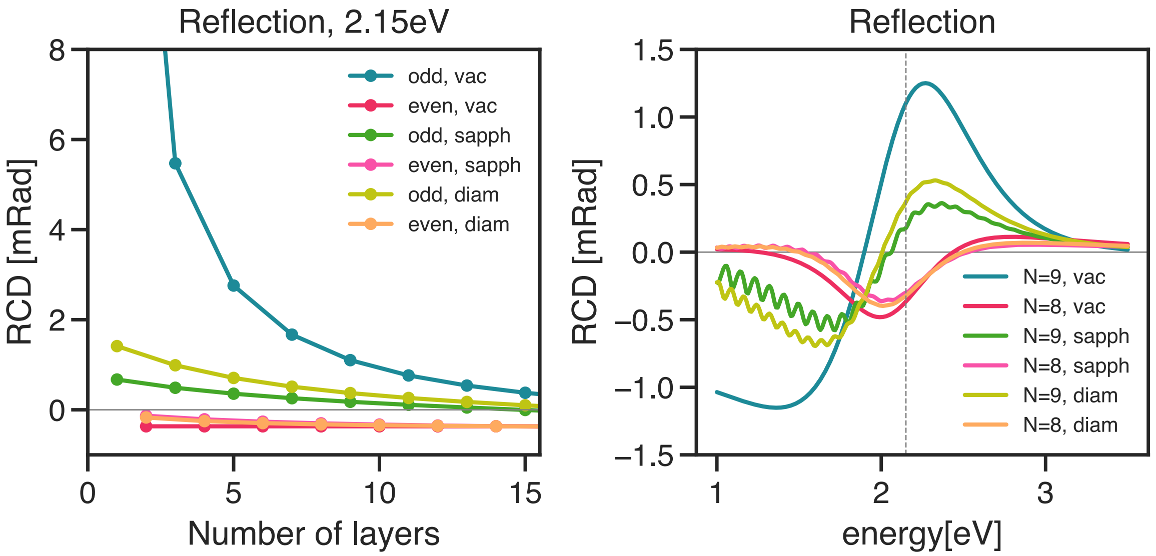

Figure S9. Comparison of RCD for samples suspended in vacuum and on two substrates, sapphire and diamond. (a) RCD as a function of layer number, for photon energy of 2.15 eV. Odd-layer RCD is significantly suppressed for samples on substrates compared to the ones suspended in vacuum. (b) RCD spectra for $N = 8$ and $N = 9$, showing that samples on substrates exhibit smaller differences in RCD magnitude for even and odd layer flakes across the spectrum. Calculation is done using the Lorentz model (Eq. 1), with parameters listed in Table S1. The sapphire substrate is modeled with an energy independent dielectric constant $\varepsilon = \varepsilon_0 \left(10 + 10^{-4} i\right)$, and the diamond with $\varepsilon = \varepsilon_0 \left(5.7 + 10^{-4} i\right)$, where we added the small imaginary components to suppress multiple reflections, which give rise to the oscillatory component of the RCD vs energy. The thickness of the substrates is taken to be 1 cm.
