# Supplementary material for: Magneto-optical Kerr effect in an A-type antiferromagnet
Source: Nat Commun. 2026 May 12;17:7364. doi: 10.1038/s41467-026-72577-4 (PMC13402694; doi:10.1038/s41467-026-72577-4)
Supplement: Supplementary file 2 — Peer Review File [file 41467_2026_72577_MOESM2_ESM.pdf]

nature portfolio

Peer Review File

Magneto-optical Kerr effect in an A-type antiferromagnet

Corresponding Author: Professor Veronika Sunko

This manuscript has been previously reviewed at another journal. This document only contains information relating to versions considered at Nature Communications.

This file contains all reviewer reports in order by version, followed by all author rebuttals in order by version.

Version 0:

Reviewer comments:

Reviewer #1

(Remarks to the Author)
The phenomenon of MOKE in antiferromagnets is of significant interest, especially given the recent focus on magnetic topological insulators. The manuscript provides a valuable advance to the magneto-optics community by directly linking the Kerr signal in an A-type AFM to the conventional layer-by-layer optics mechanism rather than exotic axion effects. The revised manuscript has addressed the reviewers' major concerns with additional data and simulations, and clarified the novelty – this is a bulk demonstration of Kerr signals. As before, I find the experimental and theoretical analyses robust, and recognize the potential significant impact. This work merits publication in Nature Communications, and I recommend minor revisions before acceptance:

1) The original title has been modified to appropriately narrow the claim of generality. However, the supplementary materials still contains the old title which has to be fixed. The word "universal" has been dropped from the title, but the abstract still says "observable MOKE should be expected in all collinear A-type AFMs". This prediction is plausible, but until other materials are measured it remains theoretical. The authors should ensure it is clear this generality comes from the theoretical model, supported by their robust demonstration, and not from experimental demonstration alone.
2) Furthermore, the abstract has been revised to delineate bulk and few-layer limits. The authors correctly answer to the Reviewer's 3 concern about MOKE on CrI_3 that bulk CrI_3 is ferromagnetic and ultrathin even-layered CrI_3 flakes were substrate-supported, so not directly relevant. Still, it may be helpful to explicitly discuss the key references for CrI_3 in the introduction or discussion (e.g. on p.13 within the paragraph starting with "The effect we observe...") of the main text, and to clarify distinctions in mechanisms of how RCD signals emerge in a symmetry-broken ultrathin film vs. inversion-symmertic bulk, to clearly position the present work against the background of previous studies.
3) Reviewer 1 raised the concern that the smaller coercive field in the AFM phase (Fig. 2a) might arise from surface oxidation or defects with residual magnetization, rather than intrinsic AFM reversal. The authors have convincingly responded to this by adding additional measurements of thick flakes from the same batch, verifying reproducibility. They also added simulations (Fig. S2) showing that switching only a surface FM layer produces an asymmetric RCD signature different from the observed. It would strengthen the manuscript if the main text explicitly refers to Fig. S2 and its conclusions as well.

Reviewer #2

(Remarks to the Author)
The authors have addressed all my concerns convincingly. It is now clear that the MOKE signal observed at zero field originates from the antiferromagnetic state, rather than a uncompensated ferromagnetic state. The study demonstrates that by varying the applied magnetic field, a transition between the two AFM states can occur within the antiferromagnetic phase. This work provides valuable insights into existing controversies in the literature. I recommend its publication in Nature Communications.

Open Access This Peer Review File is licensed under a Creative Commons Attribution 4.0 International License, which permits use, sharing, adaptation, distribution and reproduction in any medium or format, as long as you give appropriate credit to the original author(s) and the source, provide a link to the Creative Commons license, and indicate if changes were made.
In cases where reviewers are anonymous, credit should be given to 'Anonymous Referee' and the source.
The images or other third party material in this Peer Review File are included in the article's Creative Commons license, unless indicated otherwise in a credit line to the material. If material is not included in the article's Creative Commons license and your intended use is not permitted by statutory regulation or exceeds the permitted use, you will need to obtain permission directly from the copyright holder.
To view a copy of this license, visit https://creativecommons.org/licenses/by/4.0/

We thank the reviewer for the positive feedback, and the suggestions for further improvement. We address their remaining points below.

1) The original title has been modified to appropriately narrow the claim of generality. However, the supplementary materials still contains the old title which has to be fixed. The word "universal" has been dropped from the title, but the abstract still says "observable MOKE should be expected in all collinear A-type AFMs". This prediction is plausible, but until other materials are measured it remains theoretical. The authors should ensure it is clear this generality comes from the theoretical model, supported by their robust demonstration, and not from experimental demonstration alone.

We fixed the title of the SI, and amended the last sentence of the abstract: "*Our model suggests that observable MOKE should be expected in all collinear A-type AFMs with out-of-plane spin order, thus enabling optical detection of AFM domains and expanding the scope of MOKE to few-layer AFMs*."

2) Furthermore, the abstract has been revised to delineate bulk and few-layer limits. The authors correctly answer to the Reviewer's 3 concern about MOKE on CrI_3 that bulk CrI_3 is ferromagnetic and ultrathin even-layered CrI_3 flakes were substrate-supported, so not directly relevant. Still, it may be helpful to explicitly discuss the key references for CrI_3 in the introduction or discussion (e.g. on p.13 within the paragraph starting with "The effect we observe...") of the main text, and to clarify distinctions in mechanisms of how RCD signals emerge in a symmetry-broken ultrathin film vs. inversion-symmertic bulk, to clearly position the present work against the background of previous studies.

We agree that such a discussion would be helpful in principle. However, we think that there are sufficient inconsistencies in the literature regarding the behavior of thin flakes that we would rather avoid explicit discussion of specific results. For instance, even-layer flakes of CrI3 do not exhibit MOKE in Ref. [2] of our manuscript, but they do in Refs. [41] and [42]. The discrepancy has been attributed to different fabrication protocols – in Ref. [2] pristine flakes are studied, while in Refs. [41] and [42] flakes are encapsulated. However, all those measurements were performed at a single wavelength, so it is also possible that all samples exhibit RCD at some wavelength, but the spectrum is somewhat different depending on the sample geometry. We believe our results motivate future studies on thin flakes of CrI3, which will clarify these points.

3) Reviewer 1 raised the concern that the smaller coercive field in the AFM phase (Fig. 2a) might arise from surface oxidation or defects with residual magnetization, rather than intrinsic AFM reversal. The authors have convincingly responded to this by adding additional measurements of thick flakes from the same batch, verifying reproducibility. They also added simulations (Fig. S2) showing that switching only a surface FM layer produces an asymmetric RCD signature different from the observed. It would strengthen the manuscript if the main text explicitly refers to Fig. S2 and its conclusions as well.

Thank you for bringing up that this link was missing. We add the following text to page 7, just before the "Lorentz model" section: "*Further, in the SI (Fig. S2) we consider whether modified surface properties could account for this observation, and we conclude that such explanation is inconsistent with the data. This leaves us with the following conclusion: MOKE at $H=0$ arises from the bulk antiferromagnetic structure.*"

Additionally, we cited a follow up theoretical work that is now on arXiv (Ref 44 of the revised manuscript).
